# Supplementary material for: Systems genetics analysis identifies calcium-signaling defects as novel cause of congenital heart disease
Source: Genome Med. 2020 Aug 28;12:76. doi: 10.1186/s13073-020-00772-z (PMC7453558; doi:10.1186/s13073-020-00772-z)
Supplement: Supplementary file 1 — Additional file 1: Supplementary Figures and Tables. Pedigrees of 32 Danish multiplex CHD families (Fig. S1). Relatedness of the 90 individuals included in the study (Fig. S2). Homogeneity of the cohort (Fig. S3). Principle component analysis (Fig. S4). Sequencing coverage and number of reads (Fig. S5). Overview of the sequencing and data analysis processes (Fig. S6). Number of CDGs per family when all rare variants (left) or only high severity variants (right) were considered (Fig. S7). Overlap between CDGs in pairs of families (Fig. S8). Families with rare inherited variants in CDGs (Fig. S9). Overlap between the 1,785 CDGs in our families and a curated list of 829 genes known to cause CHD in mice (Fig. S10). Distribution of CHD genes across families (Fig. S11). Quantile-quantile plots (Fig. S12). Distribution of pathogenic mutations in random gene-sets (Fig. S13). Injection of sub-efficient doses of MOs and quantification of heart phenotypes in WT, controls and morphants injected with second set of MOs (Fig. S14). Efficiency of the splice blocking morpholinos (MOs) used against adcy2a, itpr1b and plcb2 (Fig. S15). Human orthologues to 829 genes known to be associated with CHD in mouse models (Table S1). A list of 144 Human CHD disease genes (Table S2). Primers used in the study (Table S3). Variants identified in known human CHD genes (Table S4). Significant protein-protein interaction clusters with at least two CDGs (Table S5). Rare calcium signaling gene variants shared among affected individuals in multiplex CHD families (Table S6). Gene ontology term enrichment of 27 genes within the cluster shown in Fig. 3a (Table S7). Replication using WES data from 714 CHD cases and 4922 controls (Table S8). [file 13073_2020_772_MOESM1_ESM.pdf]

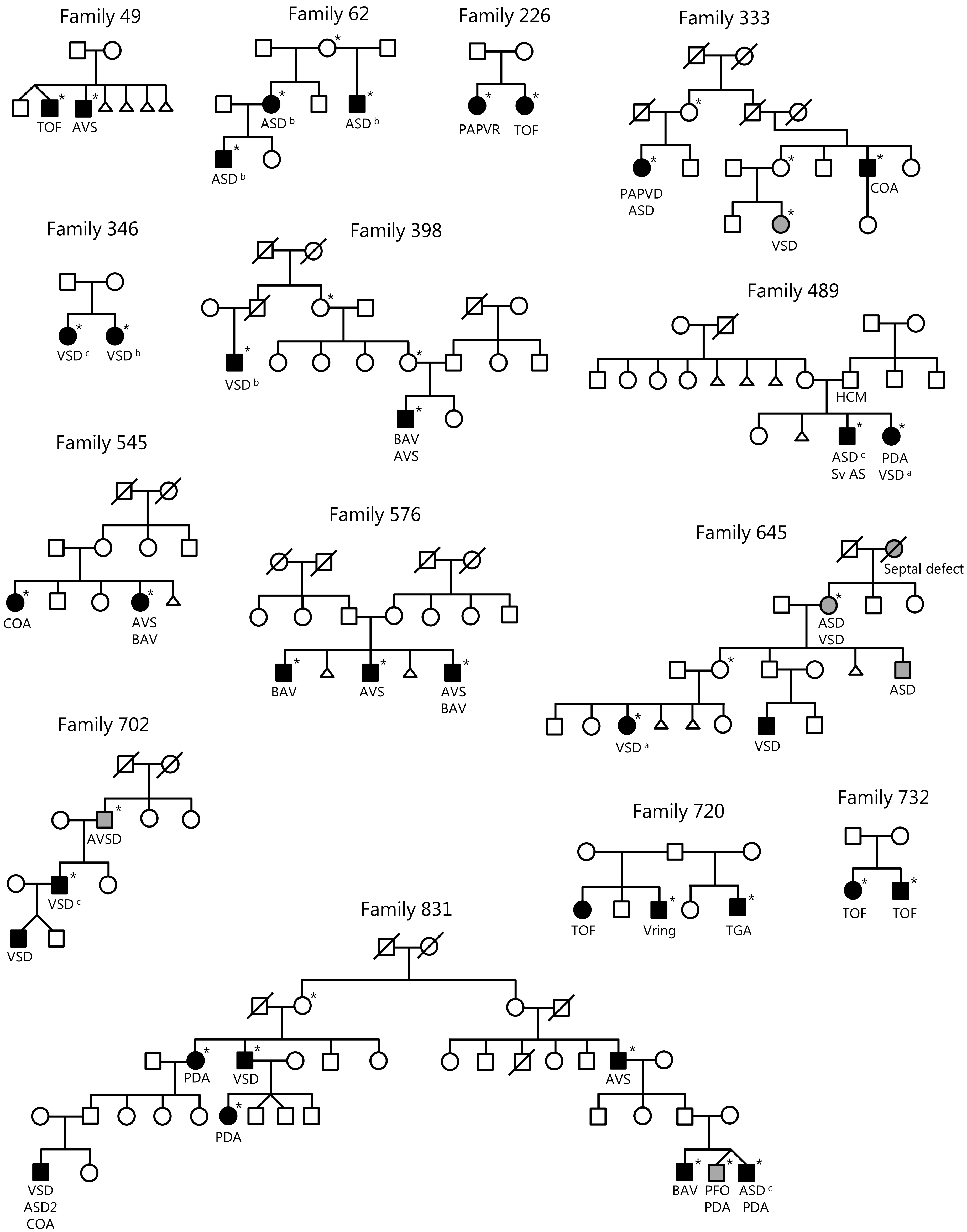
Family 49
TOF
AVS
Family 62
ASD [b]
ASD [b]
ASD [b]
Family 226
PAPVR
TOF
Family 333
PAPVD
ASD
COA
VSD
Family 346
VSD [c]
VSD [b]
Family 398
VSD [b]
BAV
AVS
Family 489
HCM
ASD [c]
Sv AS
PDA
VSD [a]
Family 545
COA
AVS
BAV
Family 576
BAV
AVS
AVS
BAV
Family 645
Septal defect
ASD
VSD
ASD
VSD [a]
VSD
Family 702
AVSD
VSD [c]
VSD
Family 720
TOF
Vring
TGA
Family 732
TOF
TOF
Family 831
PDA
VSD
AVS
PDA
VSD
ASD2
COA
BAV
PFO
PDA
ASD [c]
PDA

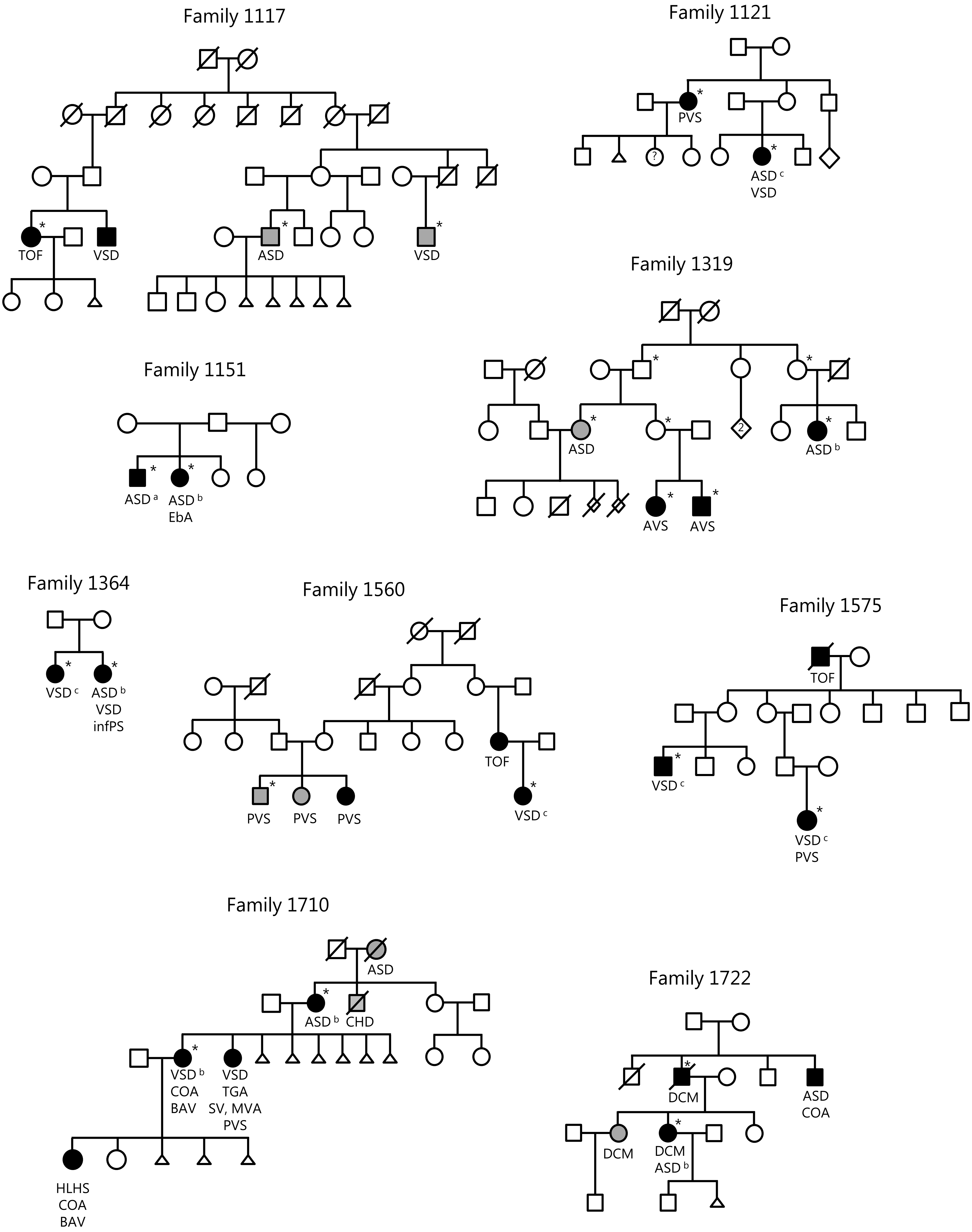
Family 1117
TOF
VSD
ASD
VSD
Family 1121
PVS
?
ASD c
VSD
Family 1319
ASD
2
ASD b
AVS
AVS
Family 1151
ASD a
ASD b
EbA
Family 1364
VSD c
ASD b
VSD
infPS
Family 1560
TOF
PVS
PVS
PVS
VSD c
Family 1575
TOF
VSD c
VSD c
PVS
Family 1710
ASD
ASD b
CHD
VSD b
COA
BAV
VSD
TGA
SV, MVA
PVS
HLHS
COA
BAV
Family 1722
DCM
ASD
COA
DCM
DCM
ASD b

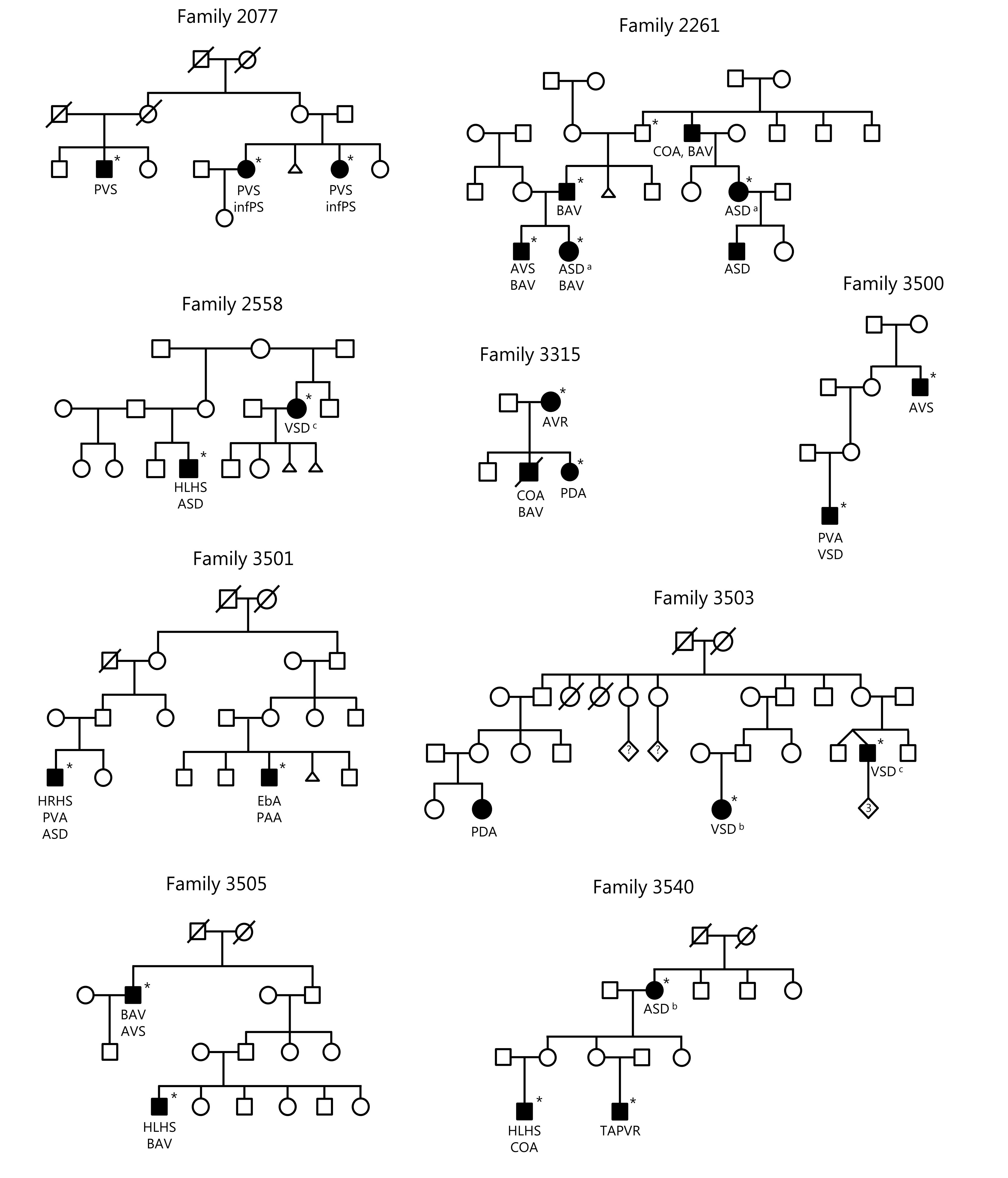

Fig S1. Pedigrees of 32 Danish multiplex CHD families. Circles: females. Squares: males. White symbols: unaffected family members. Filled symbols: affected family members. Triangles: abortion. CHD was determined by manual inspection of patient files (black) or data from DNPR/interview with family members (grey). Exome sequenced individuals are marked with asterix. The following heart defects were identified; Aortic Valve Regurgitation (AVR), Aortic Valve Stenosis (AVS), Atrial Septal Defect (ASD), Atrioventricular Septal Defect (AVSD), Bicuspid Aortic Valve(BAV), Coarctation of the Aorta (COA), Dilated Cardiomyopathy (DCM), Ebstein's Anomaly (EbA), Hypertrophic Cardiomyopathy (HCM), Hypoplastic Left Heart Syndrome (HLHS), Infundibular Pulmonary Stenosis (InfPS), Mitral Valve Atresia (MVA), Partial Anomalous Pulmonary Venous Return (PAPVR), Patent Ductus Arteriosus (PDA), Patent Foramen Ovale (PFO), Pulmonary Valve Atresia (PVA), Pulmonary Valve Stenosis (PVS), Single Ventricle (SV), Subvalvular Aortic Stenosis (SubAS), Tetralogy of Fallot (TOF), Total Anomalous Pulmonary Venous Return (TAPVR), Transposition of The Great Arteries (TGA), Vascular ring (Vring), Ventricular Septal Defect (VSD). For ASD and VSD spontanous closure (a), intervention performed (b) and no intervention performed (c) is indicated if information was available.

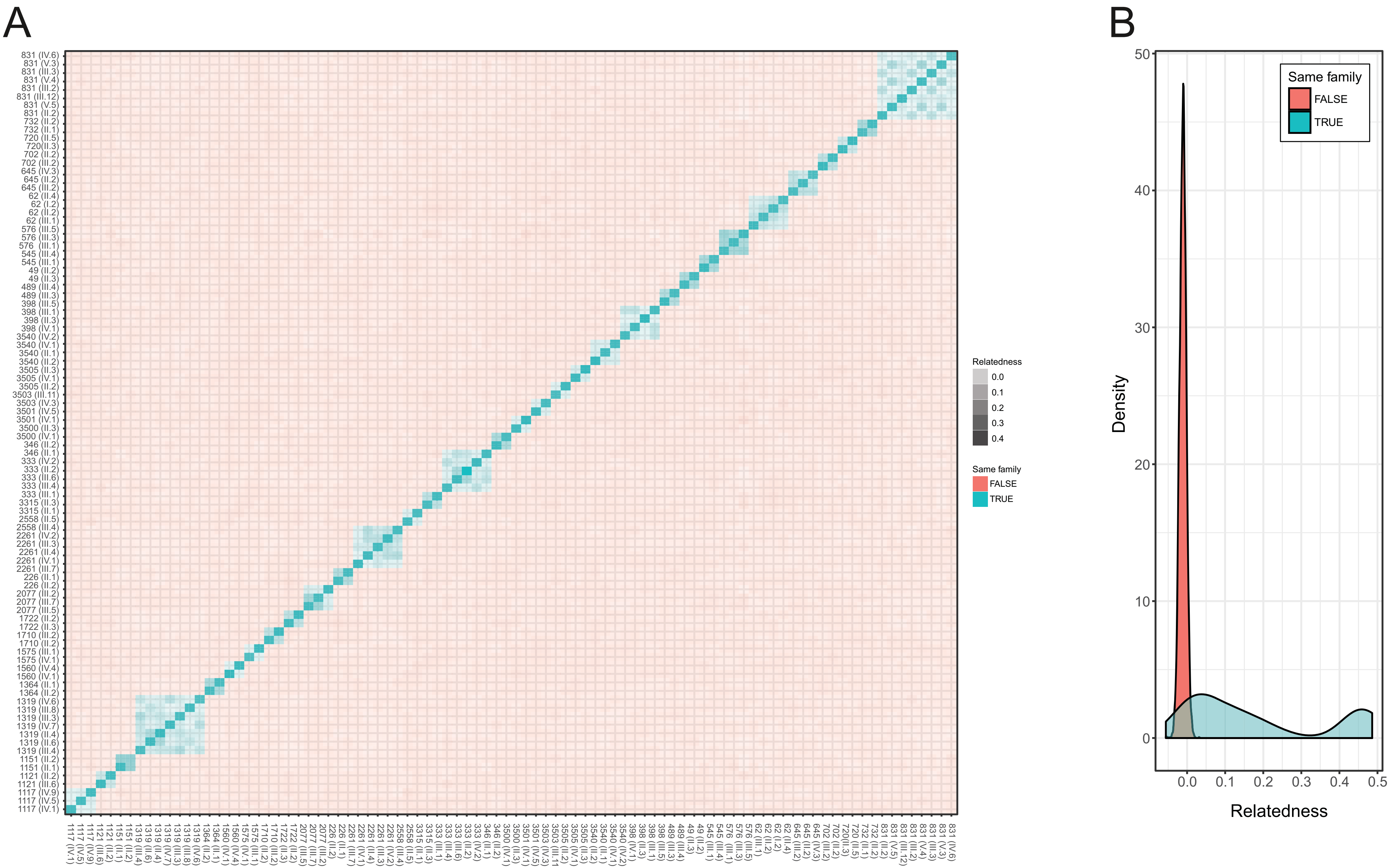

Fig. S2. Relatedness of the 90 individuals included in the study. A. Heatmap showing the relatedness score of pairs of individuals. The family number and position in the family is indicated left and below the heatmap. B. Density plot of relatedness score from the pairwise analysis. Individuals from same family is shown in blue, and individuals not from the same family is shown in red.

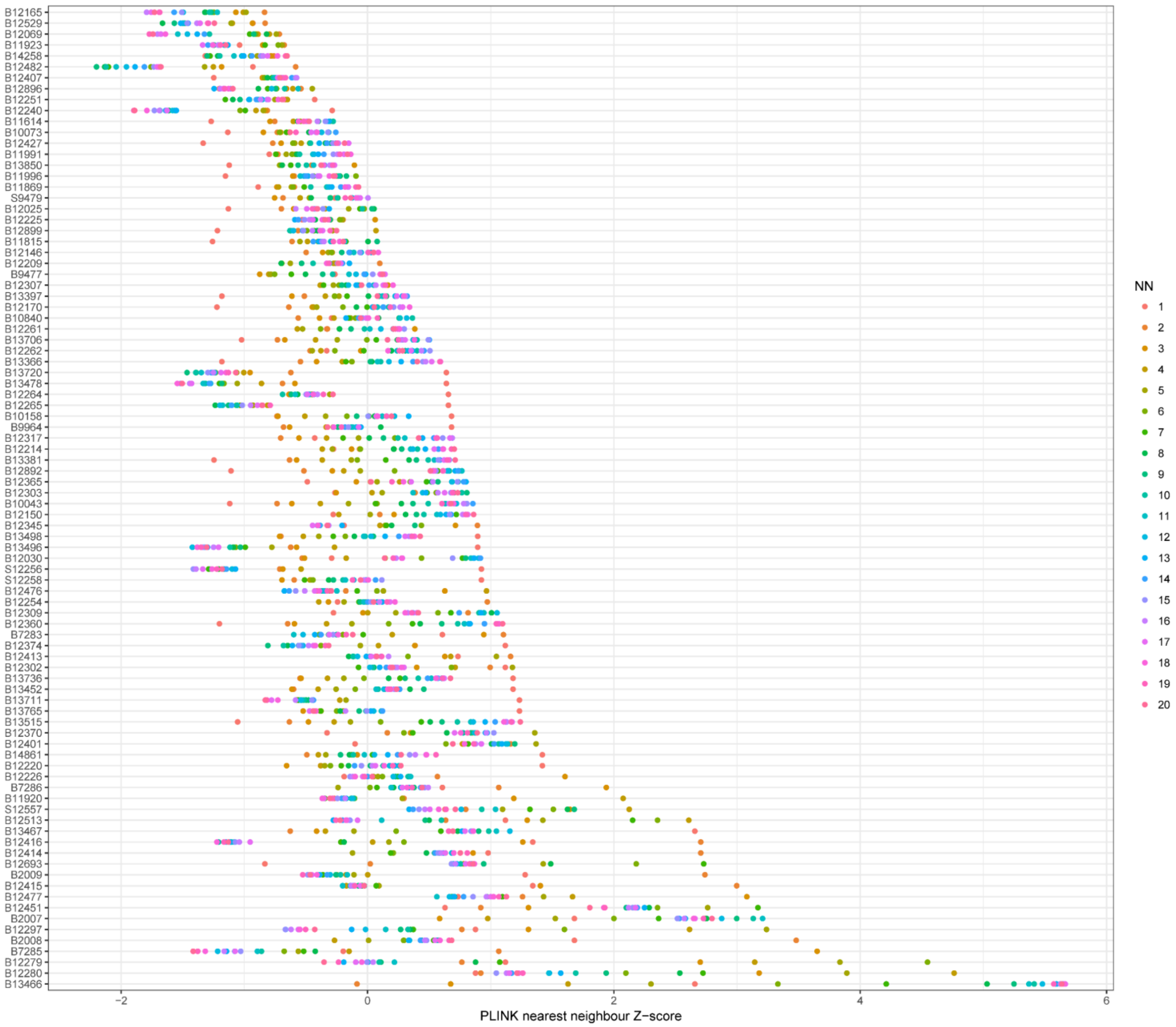

Fig. S3. Homogeneity of the cohort. Population distances from each of the 90 individuals in the cohort to the 20 nearest neighbors (NN) were calculated. The distances compared to the the mean of the population in terms of standard deviations (Z-scores) is plotted on the x-axis.

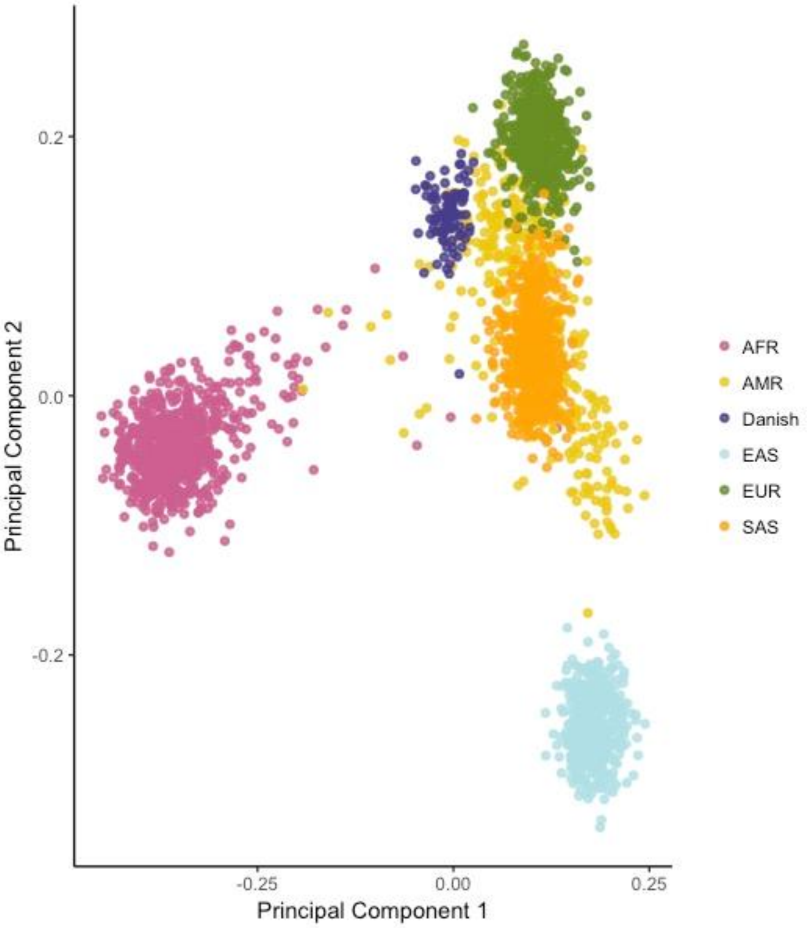

Fig. S4. Principle component analysis. The Danish samples were analyzed together with reference samples of different Super Populations from 1000 genomes (AFR= African, AMR= Admixed American, EAS= East Asian, EUR= European, SAS= South Asian).

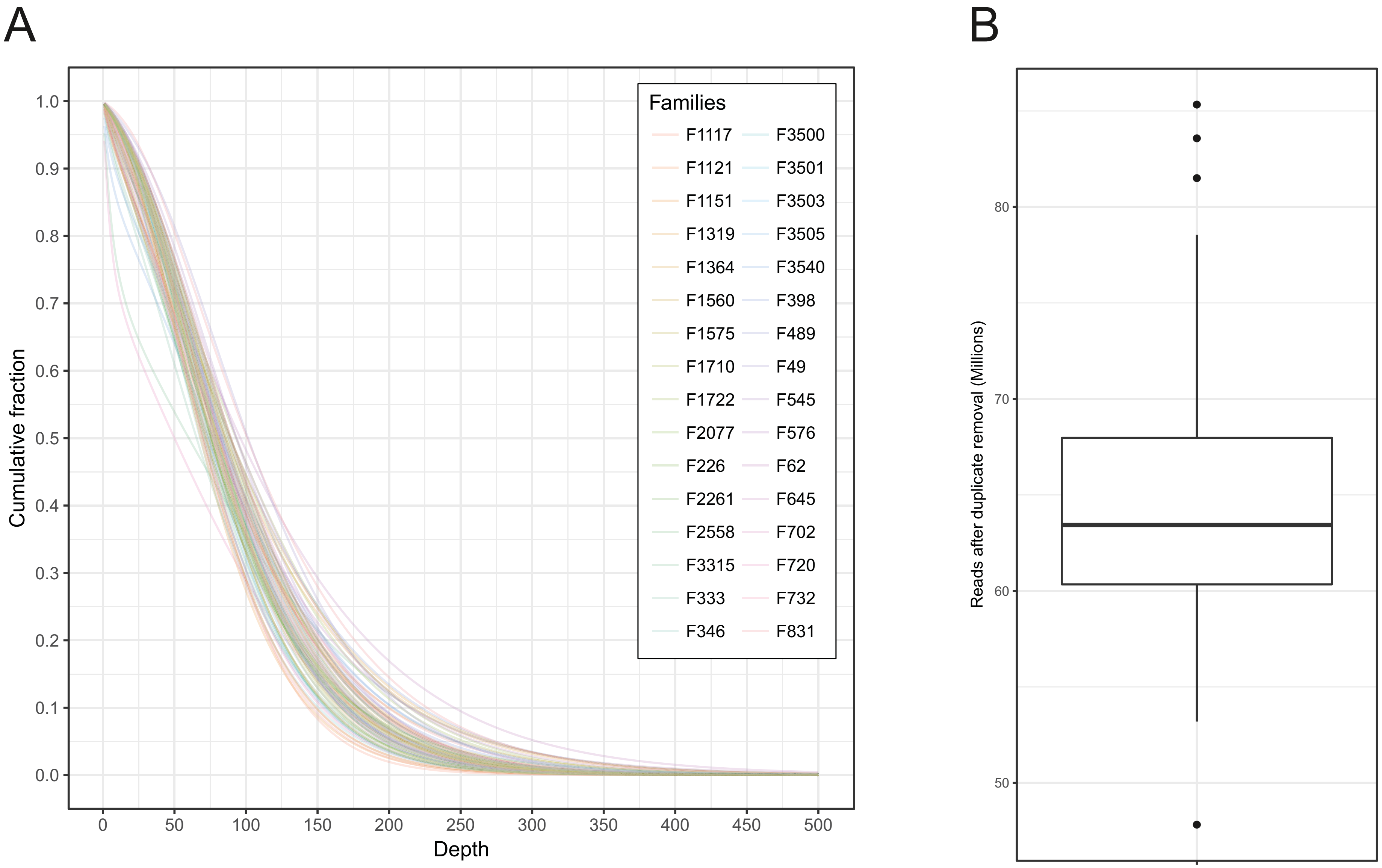

Fig. S5. Sequencing coverage and number of reads. A. Cumulative depth of sequencing per family. B. Number of reads after removal of duplicates.

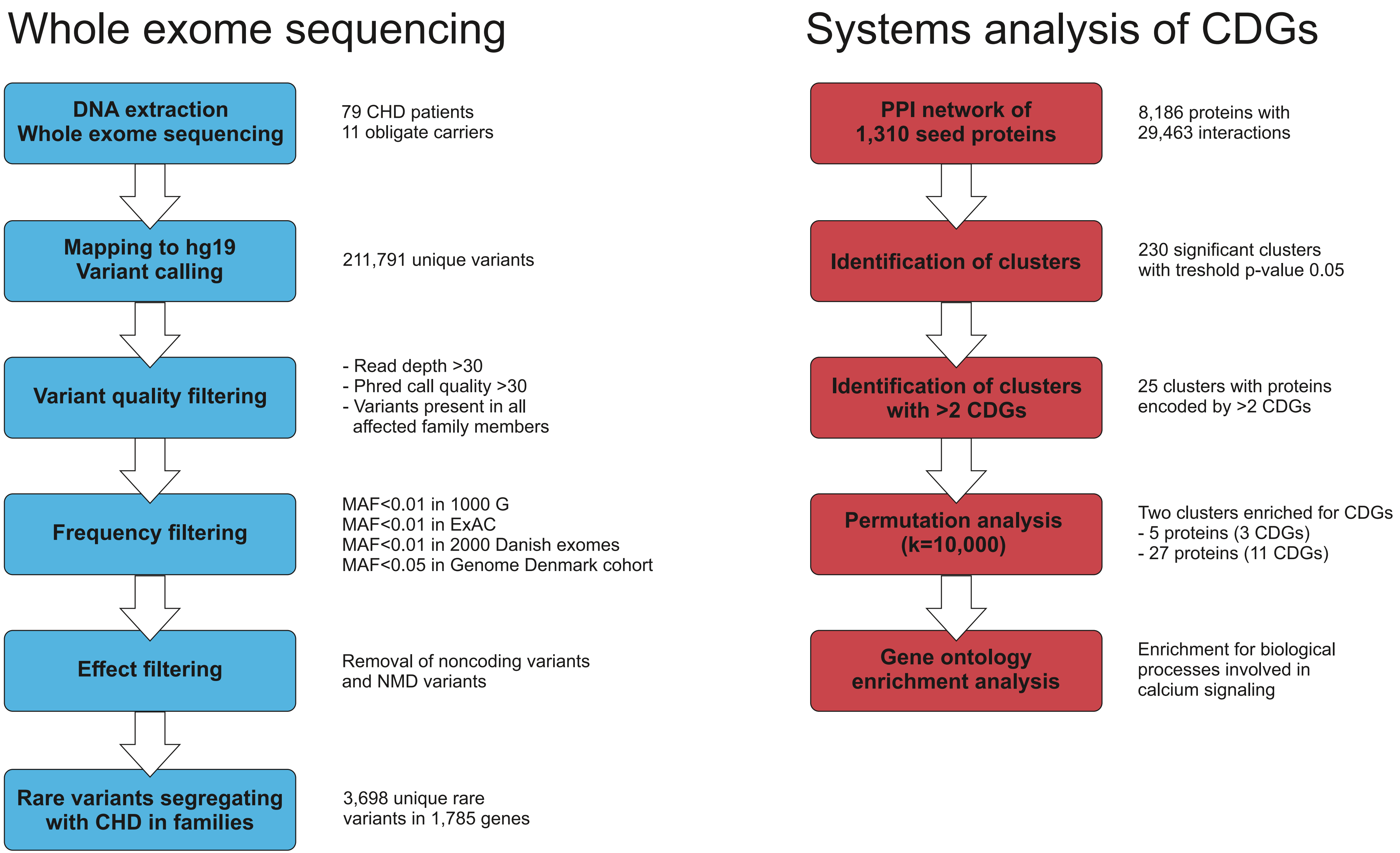

Fig. S6. Overview of the sequencing and data analysis processes.

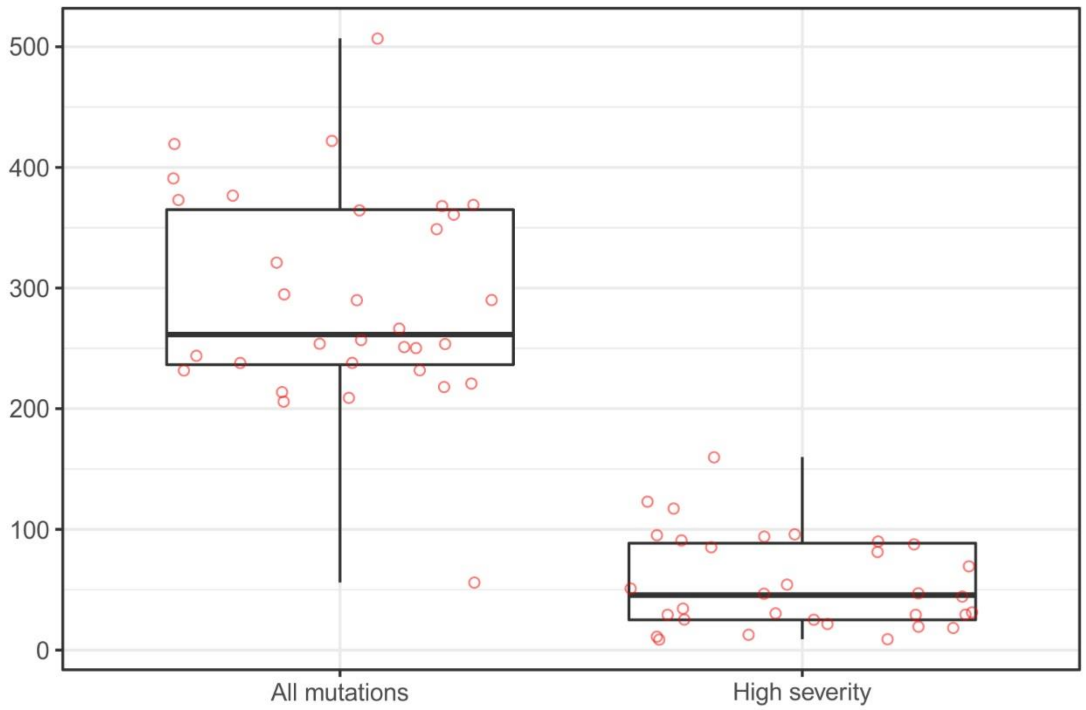

Fig. S7. Number of CDGs per family when all rare variants (left) or only high severity variants (right) were considered. High severity variants were defined as variants creating splicing defects, frameshifts, premature stop codons and missense variants predicted to be damaging by both Polyphen and SIFT.

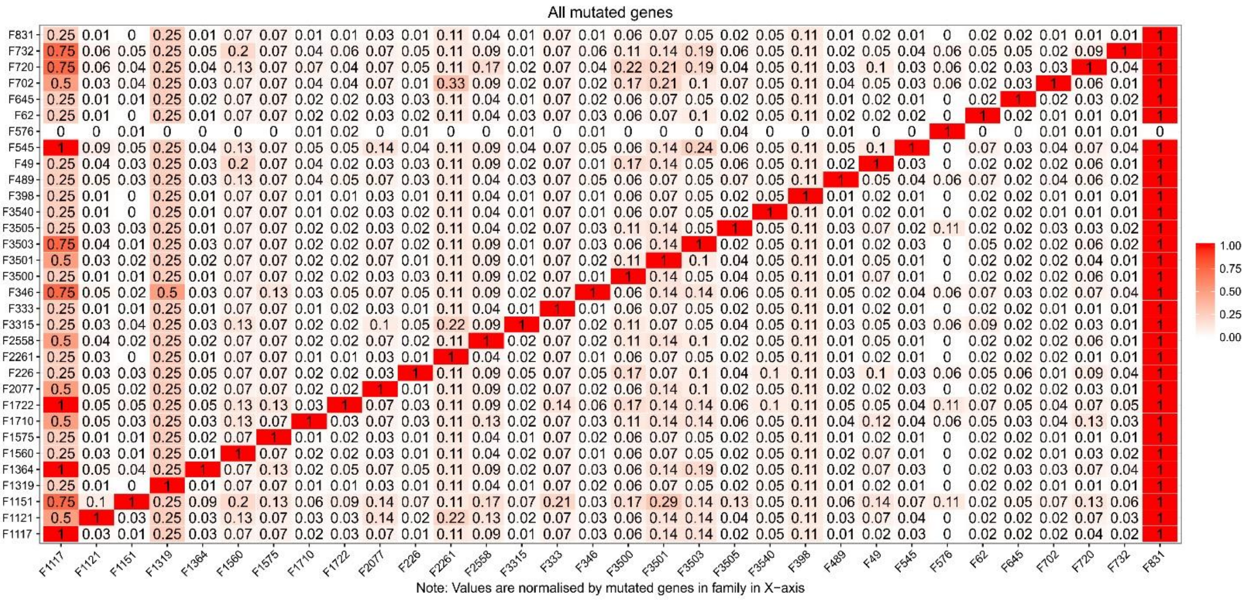

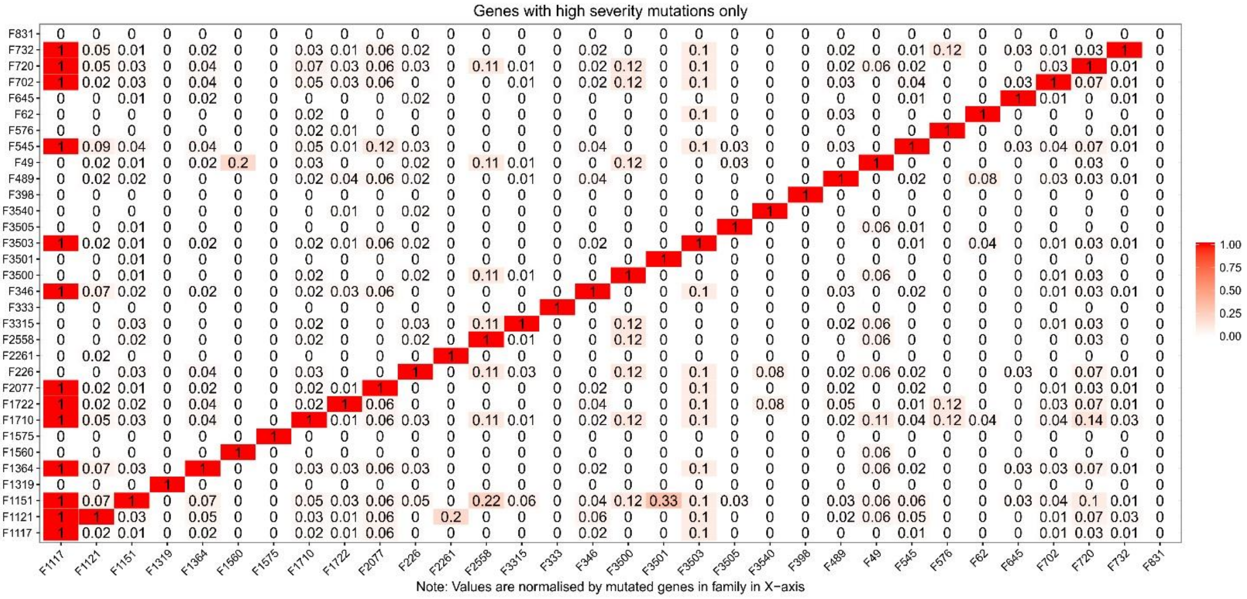

Fig. S8. Overlap between CDGs in pairs of families. The fraction of overlap (FO) between CDGs are shown for each pair of families. For example, 11% of the CDGs identified in family 49 overlaps with CDGs identified in family 2558, which is indicated by a FO value of 0.11.

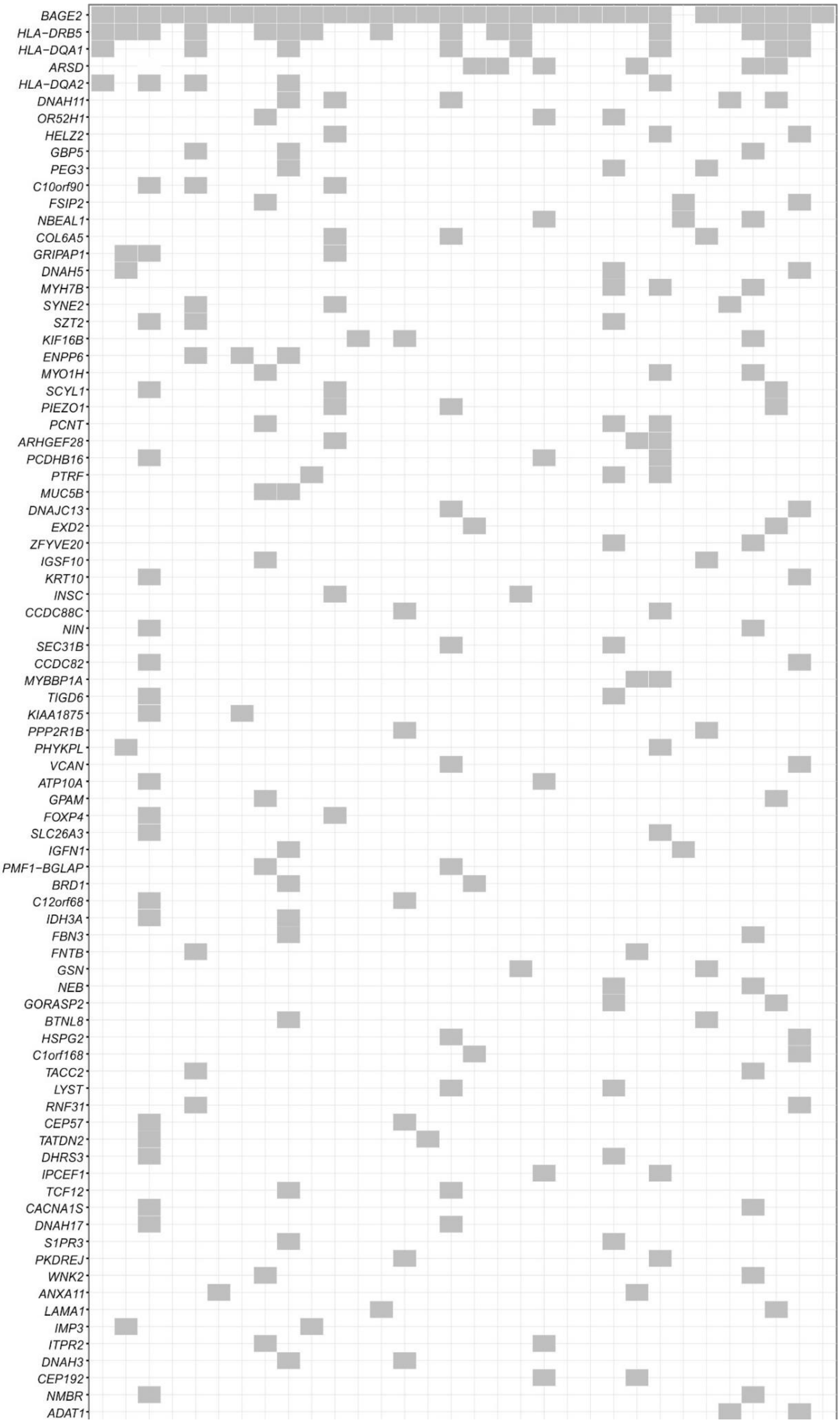
BAGE2
HLA−DRB5
HLA−DQA1
ARSD
HLA−DQA2
DNAH11
OR52H1
HELZ2
GBP5
PEG3
C10orf90
FSIP2
NBEAL1
COL6A5
GRIPAP1
DNAH5
MYH7B
SYNE2
SZT2
KIF16B
ENPP6
MYO1H
SCYL1
PIEZO1
PCNT
ARHGEF28
PCDHB16
PTRF
MUC5B
DNAJC13
EXD2
ZFYVE20
IGSF10
KRT10
INSC
CCDC88C
NIN
SEC31B
CCDC82
MYBBP1A
TIGD6
KIAA1875
PPP2R1B
PHYKPL
VCAN
ATP10A
GPAM
FOXP4
SLC26A3
IGFN1
PMF1−BGLAP
BRD1
C12orf68
IDH3A
FBN3
FNTB
GSN
NEB
GORASP2
BTNL8
HSPG2
C1orf168
TACC2
LYST
RNF31
CEP57
TATDN2
DHRS3
IPCEF1
TCF12
CACNA1S
DNAH17
S1PR3
PKDREJ
WNK2
ANXA11
LAMA1
IMP3
ITPR2
DNAH3
CEP192
NMBR
ADAT1

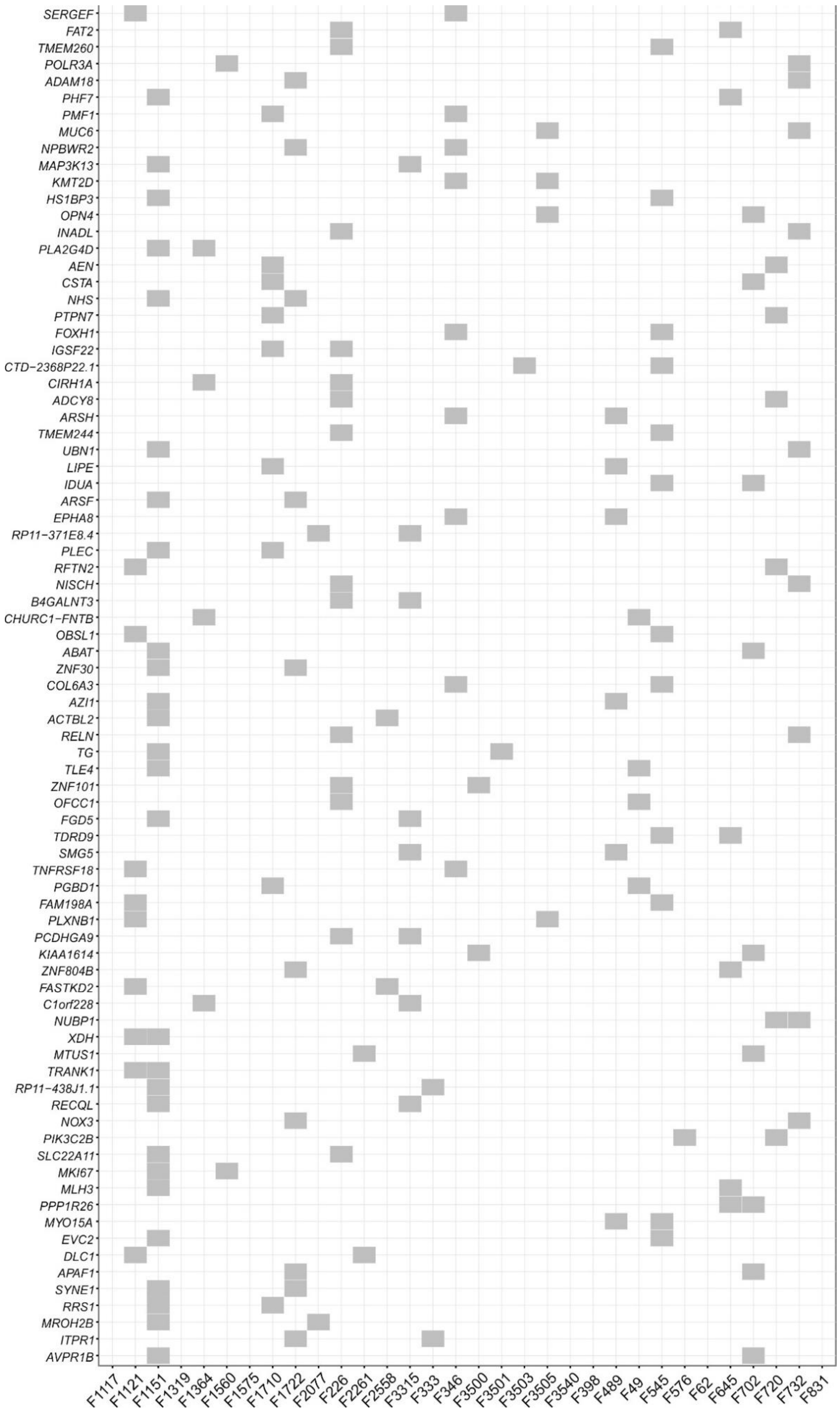

Fig. S9. Families with rare inherited variants in CDGs. Only genes mutated in two or more families are shown.

a)

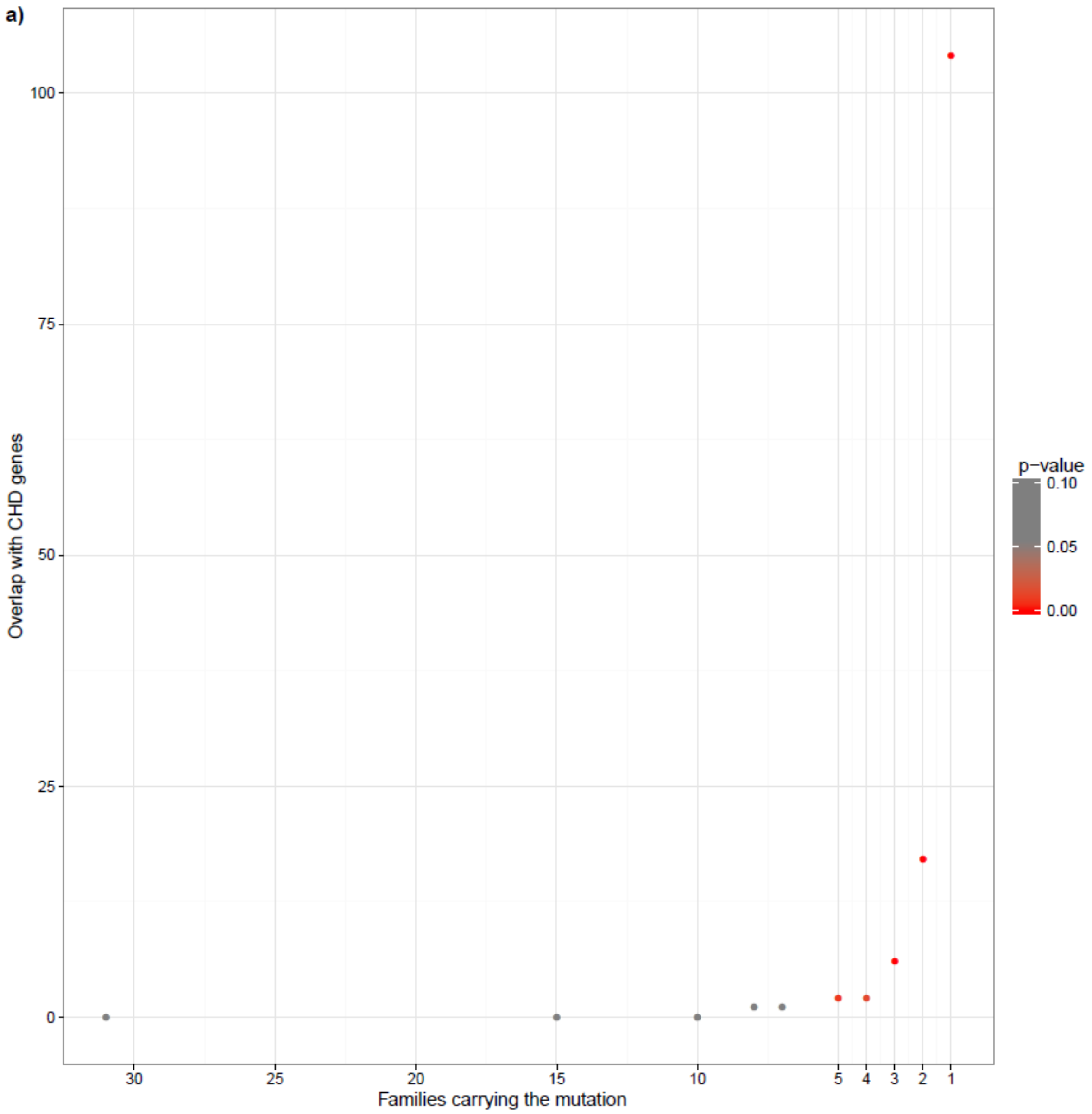

Overlap with CHD genes
100
75
50
25
0
30
25
20
15
10
5
4
3
2
1
Families carrying the mutation
p−value
0.10
0.05
0.00

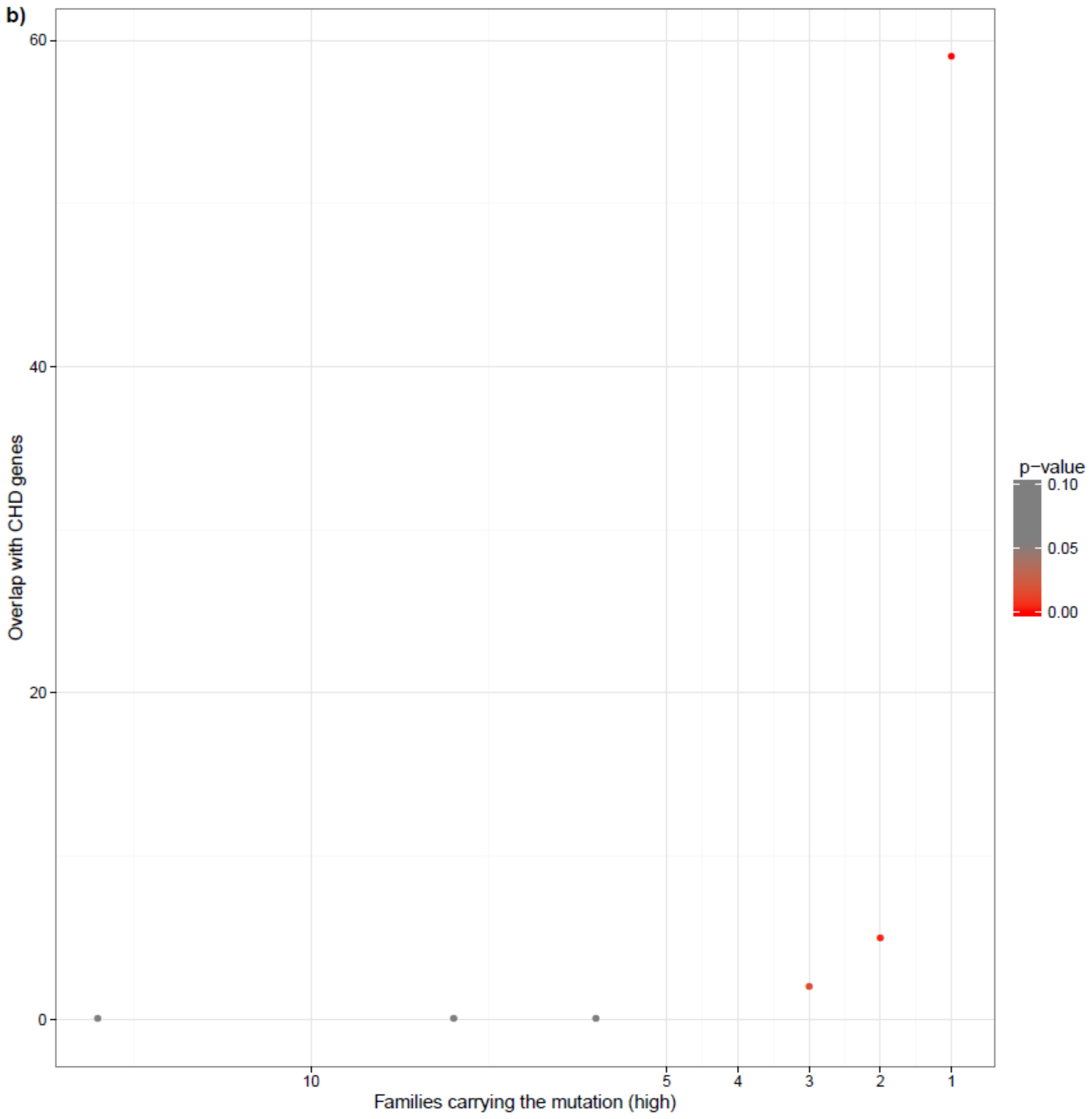

Fig. S10. Overlap between the 1,785 CDGs in our families and a curated list of 829 genes known to cause CHD in mice. A. Overlap with all 1,785 CDGs. B. Overlap with genes carrying mutations scored pathogenic by SIFT/Polyphen-2.

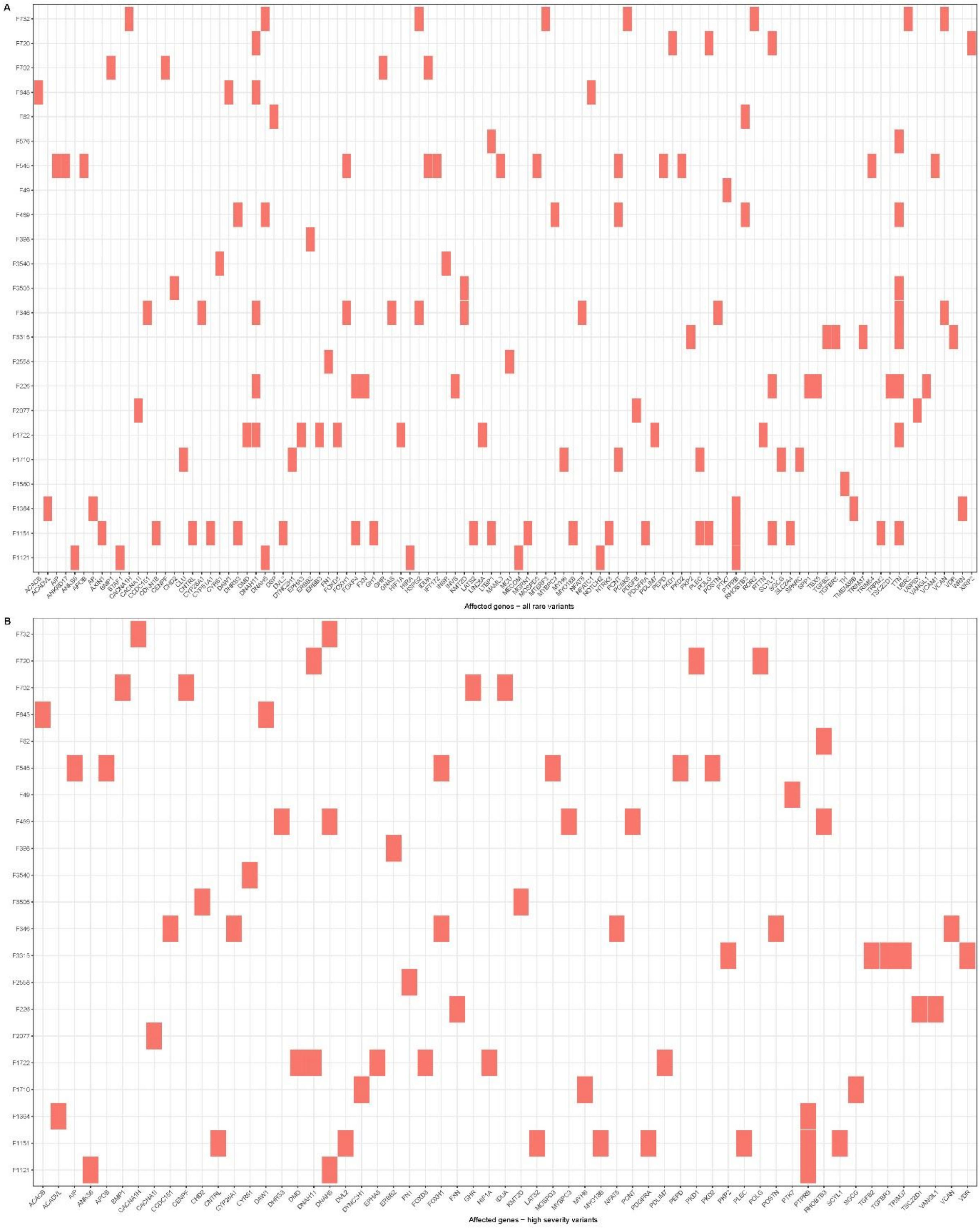

Fig. S11. Distribution of CHD genes across families. CHD genes identified in mouse models are shown here. The distribution of human CHD genes is shown in Supplemental Table 4. A. All CHD genes affected by rare variants. B. CHD genes affected by high severity variants. High severity variants were defined as variants creating splicing defects, frameshifts, premature stop codons and missense variants predicted to be damaging by both Polyphen and SIFT.

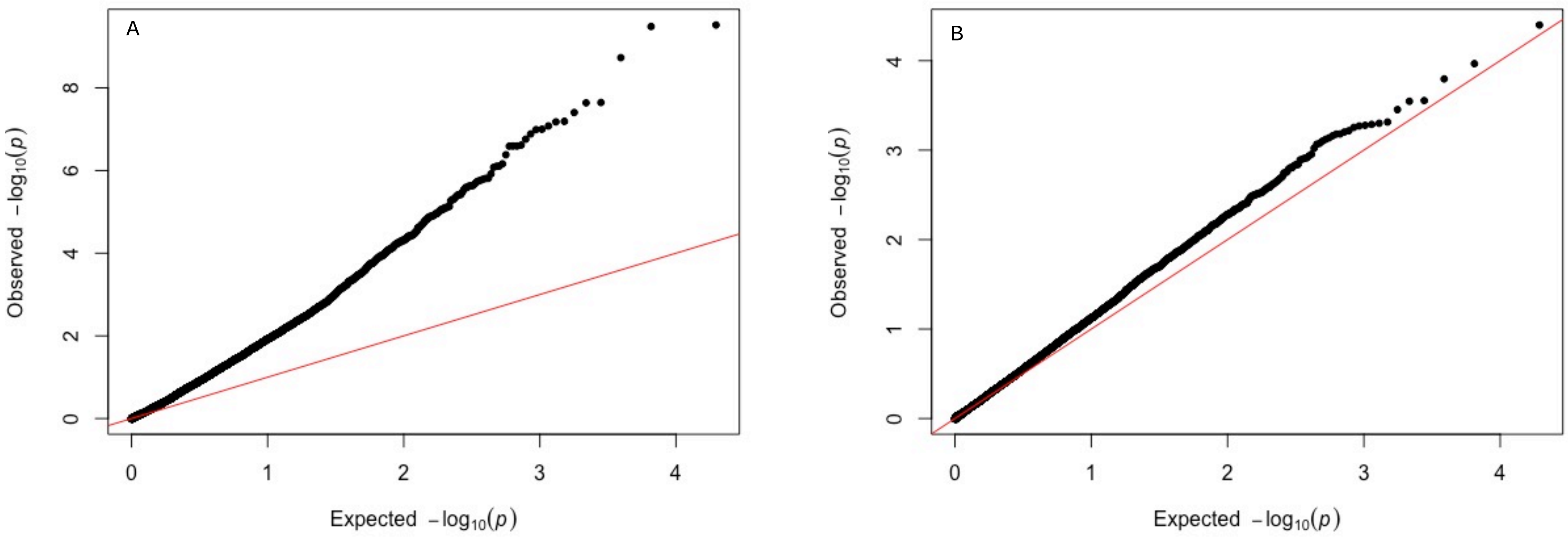

Fig. S12. Quantile-quantile plots. Plots were generated based on p-values from case control association testing (two-sided Fisher's Exact test) on the burden of synonymous mutations within 10,000 random genesets. The analysis was performed using different MAF cutoffs: A. 0.01 and B. 0.001.

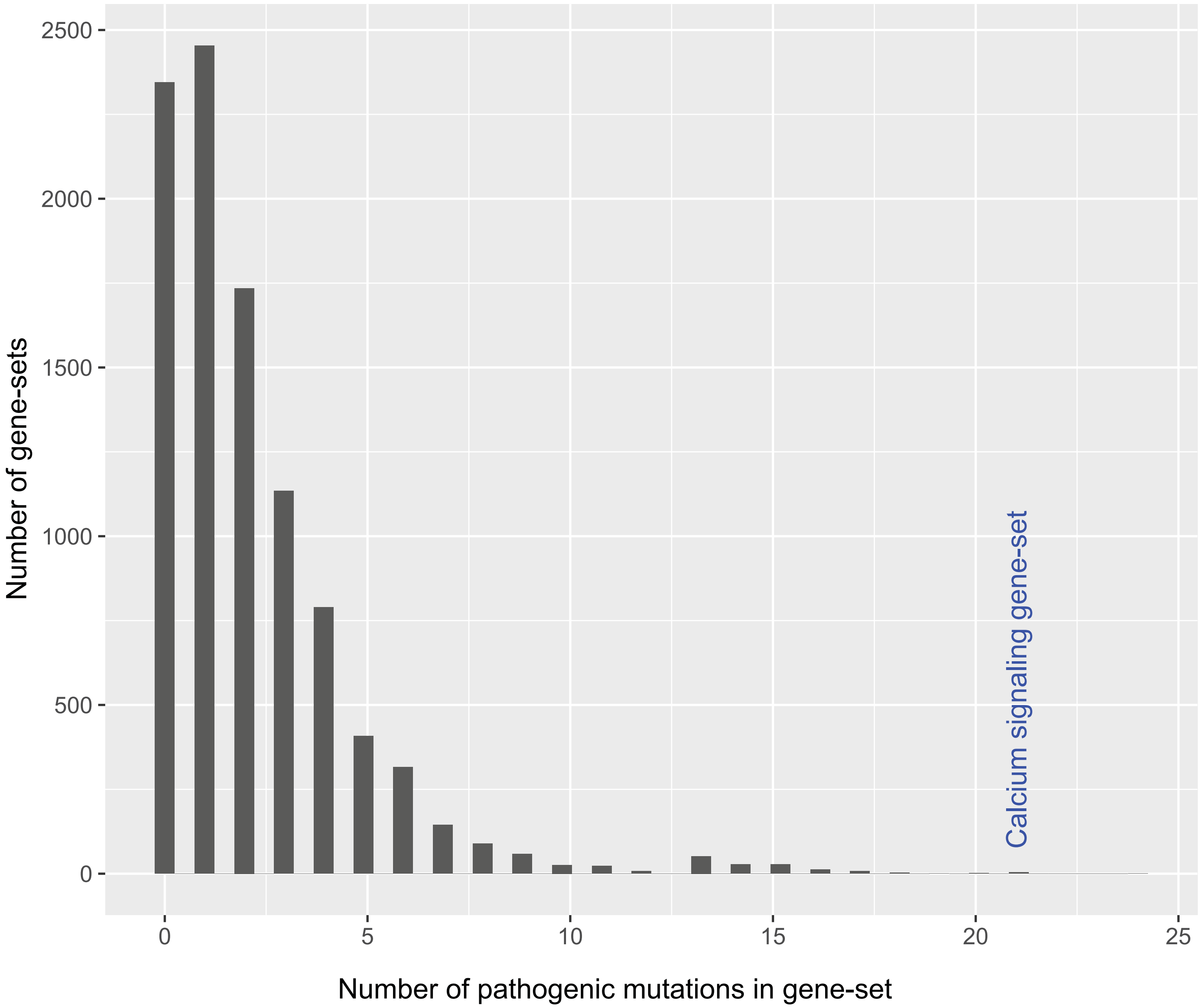

Fig. S13. Distribution of pathogenic mutations in random gene-sets. 10,000 random gene-sets of 10 genes were created. The size distribution of each set was similar to the calcium-signaling gene-set tested in the case-control study; *ADCY2* (6575 bp), *ADCY5* (7311 bp), *ITPR1* (10197 bp), *CACNA1S* (6166 bp), *CACNA1I* (6740 bp), *CACNA1H* (8208 bp), *CACNA1D* (8991 bp), *GRIA4* (5508 bp), *PLCB2* (4616 bp), and *NFAT5* (13362 bp). Pathogenic mutations were defined as mutations with MAF < 0.001 and MPC score >2.

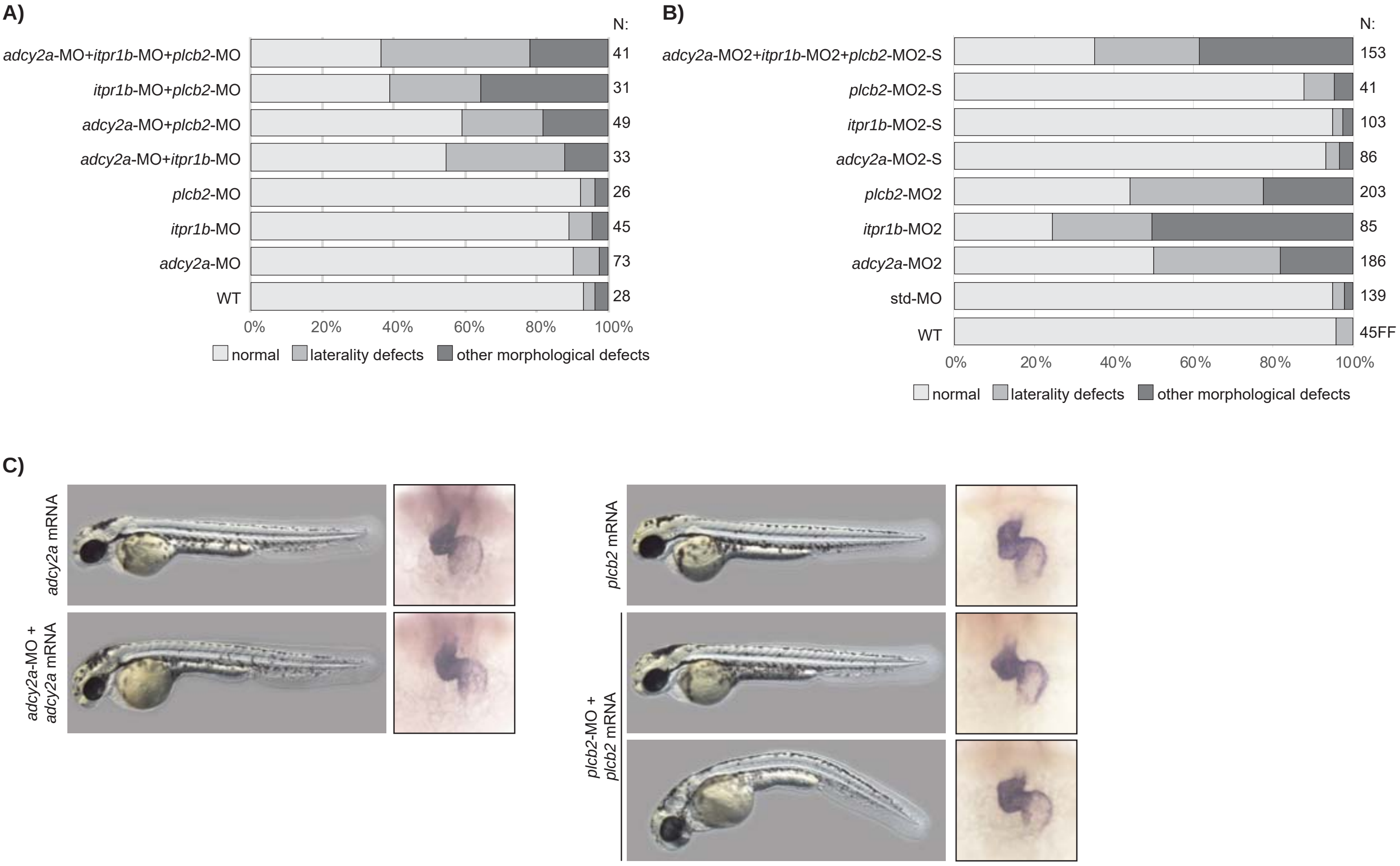

Fig. S14. A. Injection of sub-efficient doses of MOs. Note that only combinations of MOs result in significant numbers of heart defects among the embryos. B. Quantification of heart phenotypes in WT, controls and morphants injected with second set of MOs. S indicates that sub-efficient doses of MOs are injected. C. Rescue of *adcy2a* and *plcb2* knockdown. Embryos injected with *adcy2a* or *plcb2* RNA alone and in combinations with corresponding MOs, are displayed at 48 hpf. *myl7* in situ hybridization marks the heart in these embryos.

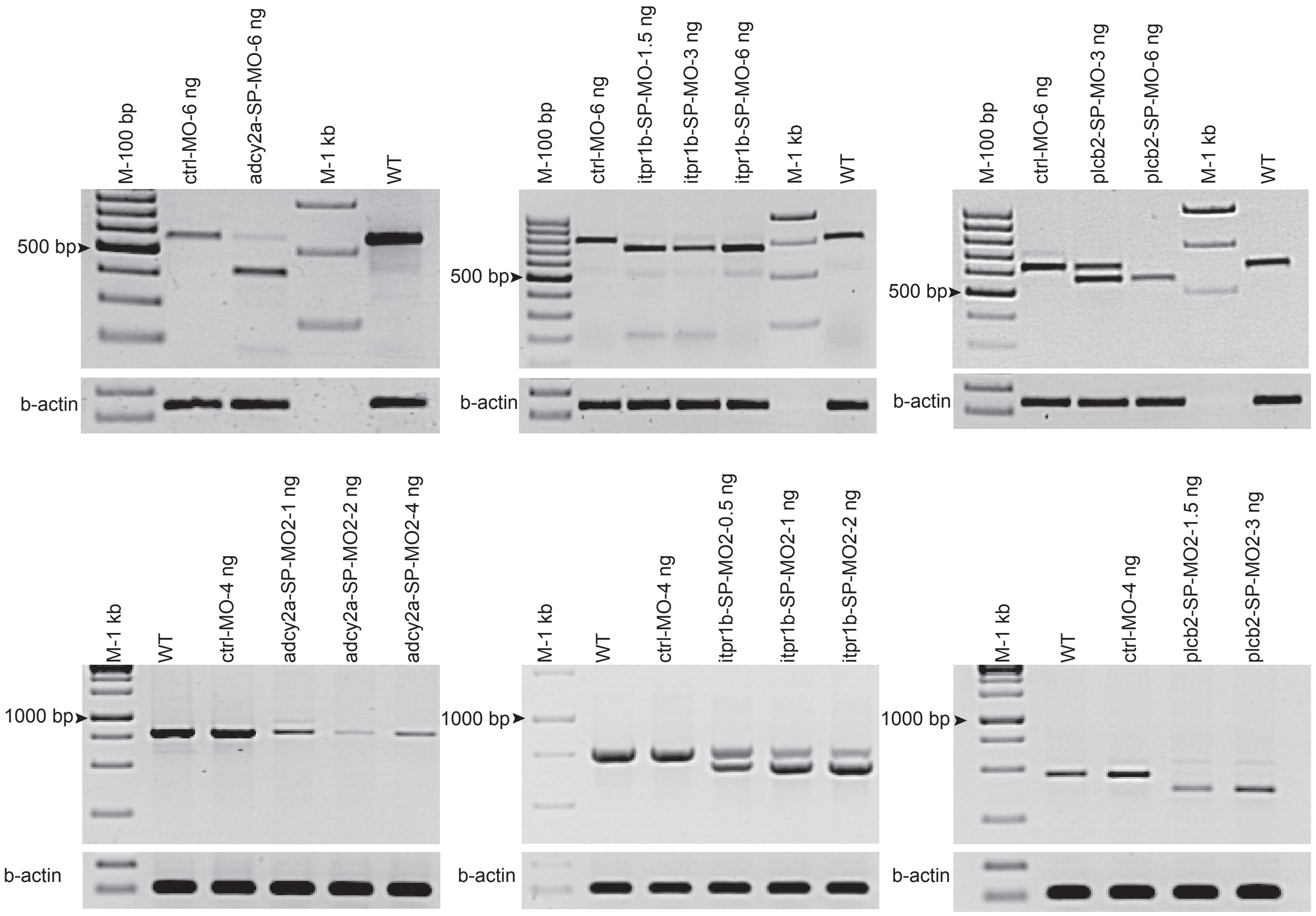

Fig. S15. Efficiency of the splice blocking morpholinos (MOs) used against *adcy2a*, *itpr1b* and *plcb2*. Upper panel shows first set of MOs and lower panel shows second set of MOs. Aberrant splicing of *adcy2a*, *itpr1b* and *plcb2* and decrease in corresponding WT mature mRNA is confirmed by RT-PCR analysis.

Table S1. Human orthologues to 829 genes known to be associated with CHD in mouse models. The list was compiled from data in the Mouse Genome Informatics database (http://www.informatics.jax.org/).

ABCA5	*CHD7*	*FGFRL1*	*INVS*	*MTERF3*	*PPARG*	*SPTBN1*
ABCB8	*CHMP5*	*FHOD3*	*IRAK1*	*MTERF4*	*PPARGC1A*	*SRF*
ABI1	*CHRD*	*FKBP1A*	*IRX3*	*MTMR12*	*PPARGC1B*	*SRSF1*
ABL1	*CHST14*	*FKBP1B*	*IRX5*	*MTO1*	*PPP1R13L*	*SRSF10*
ACACB	*CISD2*	*FLNA*	*ISL1*	*MUS81*	*PPP2R3A*	*SSBP2*
ACADM	*CITED2*	*FLRT3*	*ITCH*	*MYBPC3*	*PPP2R5C*	*SSR1*
ACADVL	*CLU*	*FMOD*	*ITGA5*	*MYCN*	*PPP3CB*	*STK39*
ACE	*CLUAP1*	*FN1*	*ITGAV*	*MYH10*	*PRDM1*	*SUFU*
ACKR3	*CNTRL*	*FOXA2*	*ITGB1*	*MYH6*	*PRDM16*	*T*
ACSL4	*COL18A1*	*FOXC1*	*ITPA*	*MYH7*	*PRDM6*	*TAB1*
ACTC1	*COL1A1*	*FOXC2*	*JAG1*	*MYL2*	*PRF1*	*TAL1*
ACVR1	*COL4A3BP*	*FOXD3*	*JARID2*	*MYL7*	*PRICKLE1*	*TBC1D32*
ACVR2A	*COMMD9*	*FOXG1*	*JMJD6*	*MYLK3*	*PRKAR1A*	*TBX1*
ACVR2B	*CRB2*	*FOXH1*	*JPH2*	*MYO10*	*PRKCI*	*TBX18*
ACVRL1	*CREBBP*	*FOXJ1*	*JUN*	*MYO18B*	*PROC*	*TBX2*
ADAM12	*CRKL*	*FOXM1*	*JUND*	*MYOD1*	*PSEN1*	*TBX20*
ADAM15	*CSNK2A1*	*FOXO1*	*JUP*	*MYOZ2*	*PSEN2*	*TBX3*
ADAM17	*CSRP2*	*FOXP1*	*KAT6A*	*NACA*	*PSKH1*	*TBX5*
ADAM19	*CSRP3*	*FOXP4*	*KCNH2*	*NCKAP1*	*PTCD2*	*TBX6*
ADAM9	*CST9*	*FOXQ1*	*KCTD10*	*NCOA6*	*PTEN*	*TCF21*
ADAMTS6	*CTBP2*	*FRAS1*	*KDM1A*	*NCOR2*	*PTGS2*	*TCTN2*
ADGRG6	*CTNNA3*	*FREM2*	*KDM2A*	*NCSTN*	*PTK2*	*TDG*
ADM	*CTNNB1*	*FRS2*	*KDM4A*	*NDST1*	*PTK7*	*TEAD1*
ADRA1A	*CXADR*	*FSTL1*	*KDM6A*	*NEGR1*	*PTPN11*	*TEAD2*
ADRA1B	*CXCL12*	*FURIN*	*KDR*	*NEK6*	*PTPRB*	*TEK*
AGT	*CXCR4*	*FUS*	*KIF3A*	*NEK8*	*PTPRJ*	*TERC*
AGTR1	*CYBB*	*FUZ*	*KIF3B*	*NF1*	*RAC1*	*TFAP2A*
AGTR2	*CYBRD1*	*FXN*	*KIF7*	*NFAT5*	*RAF1*	*TFB1M*
AIFM1	*CYP11B2*	*FXR1*	*KIFAP3*	*NFATC1*	*RAMP2*	*TGFB1*
AIP	*CYP26A1*	*FZD1*	*KISS1R*	*NFATC3*	*RARA*	*TGFB2*
AKAP12	*CYP27B1*	*FZD2*	*KLF15*	*NFATC4*	*RARB*	*TGFBR2*
AKAP13	*CYP51A1*	*FZD7*	*KLF2*	*NGF*	*RARG*	*TGFBR3*
AKAP6	*CYR61*	*GAA*	*KLF3*	*NIPBL*	*RB1*	*TGIF1*
AKT1	*DAAM1*	*GAB1*	*KLF5*	*NKX2-5*	*RB1CC1*	*TGIF2*
AKT3	*DAG1*	*GAB2*	*KLHL40*	*NKX2-6*	*RBL2*	*TGM2*
ALDH1A2	*DAGLA*	*GAS1*	*KMT2B*	*NODAL*	*RBM20*	*TH*
ALG13	*DAND5*	*GATA4*	*KMT2D*	*NOG*	*RBP4*	*THBS1*
ALPK3	*DAW1*	*GATA5*	*KRAS*	*NOS1*	*RBPJ*	*THRA*
AMN	*DCHS1*	*GATA6*	*KRT19*	*NOS3*	*RCAN1*	*TIE1*
ANGPT1	*DCTN5*	*GBE1*	*LAMA4*	*NOTCH1*	*RCE1*	*TK2*

ANKRD17	*DDR2*	*GBX2*	*LAMA5*	*NOTCH2*	*RDH10*	*TKT*
ANKS6	*DDX11*	*GDF1*	*LARGE1*	*NOV*	*RERE*	*TLL1*
AP2B1	*DDX3X*	*GH1*	*LATS2*	*NPPB*	*RFX3*	*TLR2*
AP4E1	*DEDD*	*GHR*	*LBX1*	*NPR1*	*RHBDF1*	*TMED2*
APC	*DES*	*GHRHR*	*LDB3*	*NPRL3*	*RHBDF2*	*TMEM100*
APOB	*DHRS3*	*GIPC1*	*LDLR*	*NR1D2*	*RHEB*	*TMEM106B*
APOE	*DICER1*	*GJA1*	*LEFTY1*	*NR1H3*	*RHOBTB3*	*TMEM38A*
AR	*DISP1*	*GJA5*	*LEFTY2*	*NR2F2*	*RIC8B*	*TMEM38B*
ARID1A	*DLL1*	*GJC1*	*LEMD2*	*NR3C1*	*RIPPLY3*	*TMEM67*
ARID2	*DLL4*	*GNA11*	*LEP*	*NR3C2*	*RNF4*	*TMOD1*
ARMC4	*DMD*	*GNAQ*	*LEPR*	*NRG1*	*ROBO1*	*TMSB4X*
ARNTL	*DNAAF2*	*GNAS*	*LHCGR*	*NRP1*	*ROR1*	*TNNI3*
ARSB	*DNAAF3*	*GNG5*	*LHX1*	*NRP2*	*ROR2*	*TNNT2*
ASL	*DNAH11*	*GPC3*	*LIG3*	*NTF3*	*RPGRIP1L*	*TP53*
ATE1	*DNAH5*	*GRIN2D*	*LIMS1*	*NTRK3*	*RPS6KA2*	*TREX1*
ATF2	*DNAI1*	*GRK2*	*LIMS2*	*NXN*	*RSPO3*	*TRIM37*
ATF7	*DNAJA3*	*GSC*	*LIN28A*	*OFD1*	*RTTN*	*TRIM54*
ATG5	*DNASE1L2*	*GTF2I*	*LMNA*	*OPA3*	*RXRA*	*TRIM55*
ATMIN	*DNM1L*	*GTF2IRD1*	*LMOD2*	*OSR1*	*RXRB*	*TRIM63*
ATP2A2	*DNM2*	*GYS1*	*LOXL2*	*OTX2*	*RYR1*	*TRIP11*
AXIN1	*DNMT3B*	*GYS2*	*LRP1*	*OVOL2*	*RYR2*	*TRPM2*
AXIN2	*DOCK1*	*HADHB*	*LRP2*	*PAK1*	*SALL1*	*TSC1*
B9D1	*DOT1L*	*HAND1*	*LTBP1*	*PAK4*	*SALL4*	*TSC22D1*
BAG3	*DRC1*	*HAND2*	*LTBP4*	*PARD3*	*SCN5A*	*TTBK2*
BAZ1B	*DSP*	*HAS2*	*LUM*	*PARVA*	*SCN8A*	*TTC7A*
BBX	*DTNA*	*HBEGF*	*LUZP1*	*PATZ1*	*SCYL1*	*TTN*
BCAR1	*DUSP8*	*HCCS*	*LY6E*	*PAX3*	*SEC24B*	*TXNRD2*
BCL6	*DVL1*	*HDAC2*	*MAML1*	*PAXIP1*	*SEMA3C*	*UBE2U*
BCOR	*DVL2*	*HDAC5*	*MAML3*	*PBRM1*	*SEMA3D*	*UBE4B*
BICC1	*DVL3*	*HDAC9*	*MAP1S*	*PCNT*	*SENP2*	*UBP1*
BIN1	*DYNC2H1*	*HECTD1*	*MAP2K1*	*PCSK5*	*SGCB*	*UBR1*
BMP1	*DYNC2LI1*	*HECTD3*	*MAP2K2*	*PCSK6*	*SGCD*	*UBR2*
BMP10	*DYNLL1*	*HEG1*	*MAP2K5*	*PDCD10*	*SGCG*	*UBR4*
BMP2	*DYRK1A*	*HERC4*	*MAP3K3*	*PDGFA*	*SGPL1*	*USP12*
BMP4	*DYX1C1*	*HEXIM1*	*MAP3K7*	*PDGFB*	*SH3PXD2B*	*USP24*
BMPR1A	*ECE1*	*HEY1*	*MAPK1*	*PDGFC*	*SHC1*	*USP9X*
BMPR2	*ECE2*	*HEY2*	*MAPK11*	*PDGFRA*	*SHH*	*UTF1*
BRAF	*EDN1*	*HEYL*	*MAPK14*	*PDGFRB*	*SHOC2*	*UTRN*
BTAF1	*EDNRA*	*HGS*	*MAPK3*	*PDHA1*	*SHOX2*	*UVRAG*
BTC	*EDNRB*	*HHEX*	*MAPK6*	*PDLIM3*	*SIRT1*	*VANGL1*
C2CD3	*EFNA1*	*HIC2*	*MAPK7*	*PDLIM7*	*SIRT7*	*VANGL2*
CACNA1C	*EFNB2*	*HIF1A*	*MARVELD2*	*PDPK1*	*SIX1*	*VAV2*
CACNA1H	*EGFR*	*HIF3A*	*MATR3*	*PDPN*	*SLC22A8*	*VAV3*

CACNA1I	*EGLN1*	*HIRA*	*MB*	*PDS5A*	*SLC2A4*	*VCAM1*
CACNB2	*EGLN3*	*HMOX1*	*MBTD1*	*PDS5B*	*SLC38A10*	*VCAN*
CALCRL	*EGR2*	*HOPX*	*MCU*	*PELI1*	*SLC39A4*	*VCL*
CALR	*EHMT1*	*HOXA1*	*MDM2*	*PEPD*	*SLC4A1*	*VDR*
CAPNS1	*EIF2B5*	*HOXA3*	*MECOM*	*PHC1*	*SLC6A6*	*VEGFA*
CASP8	*ELN*	*HOXB4*	*MED1*	*PHIP*	*SLC8A1*	*VEGFB*
CASQ2	*EMC10*	*HPRT1*	*MED12*	*PIBF1*	*SLC9A1*	*VPS54*
CAV1	*EMD*	*HRAS*	*MED24*	*PIFO*	*SLIT3*	*WASF2*
CAV3	*ENG*	*HSD11B2*	*MED30*	*PIGV*	*SMAD1*	*WDPCP*
CC2D2A	*EP300*	*HSD17B7*	*MEF2C*	*PIK3CA*	*SMAD2*	*WDR1*
CCDC124	*EPHA3*	*HSPB11*	*MEF2D*	*PIK3CB*	*SMAD3*	*WDR35*
CCDC151	*EPHB4*	*HSPB8*	*MEGF8*	*PIK3CG*	*SMAD4*	*WHSC1*
CCDC160	*EPO*	*HSPG2*	*MEIS1*	*PIK3R1*	*SMAD5*	*WNK1*
CCDC39	*EPOR*	*HTR2B*	*MEN1*	*PIK3R2*	*SMAD6*	*WNT11*
CCM2	*ERBB2*	*HTRA2*	*MEOX2*	*PIKFYVE*	*SMAD7*	*WNT3A*
CCM2L	*ERBB3*	*HUWE1*	*MESP1*	*PINK1*	*SMARCA4*	*WNT5A*
CCND1	*ERBB4*	*IDUA*	*MEST*	*PIP5K1C*	*SMG1*	*WRN*
CCND2	*ESR1*	*IER3*	*MFN2*	*PITX2*	*SMG9*	*WT1*
CCND3	*ESR2*	*IFT122*	*MFSD8*	*PKD1*	*SMN1*	*WWTR1*
CCNE1	*EYA1*	*IFT140*	*MGAT1*	*PKD2*	*SMO*	*XBP1*
CCNE2	*EZH2*	*IFT172*	*MGP*	*PKP2*	*SMYD1*	*XIRP1*
CDC73	*F11*	*IFT27*	*MGRN1*	*PLA2G4A*	*SNAI1*	*XIRP2*
CDH2	*F2R*	*IFT46*	*MIB1*	*PLAGL1*	*SNAI2*	*YAP1*
CDH5	*FADD*	*IFT57*	*MIXL1*	*PLCE1*	*SNX17*	*YWHAE*
CDK2	*FAT4*	*IFT74*	*MKL1*	*PLEC*	*SNX27*	*ZBTB14*
CDK4	*FBLN1*	*IFT88*	*MKL2*	*PLN*	*SOD1*	*ZFP36L1*
CDK6	*FBN1*	*IGF1R*	*MKS1*	*PLVAP*	*SOD2*	*ZFPM1*
CDKN1A	*FENDRR*	*IGF2*	*MMP21*	*PLXND1*	*SOS1*	*ZFPM2*
CDKN1B	*FES*	*IGF2R*	*MMP9*	*PNN*	*SOX11*	*ZIC3*
CENPF	*FGF10*	*IGFBP2*	*MORF4L1*	*PNPLA2*	*SOX12*	*ZNF366*
CENPJ	*FGF16*	*IGHMBP2*	*MOSPD3*	*POFUT1*	*SOX4*	*ZNF521*
CEP290	*FGF2*	*IKBKAP*	*MPZL3*	*POGLUT1*	*SOX9*	*ZSCAN10*
CFC1	*FGF8*	*IL17RD*	*MSX1*	*POLG*	*SPARC*	
CFL1	*FGF9*	*IL6*	*MSX2*	*POR*	*SPP1*	
CFLAR	*FGFR1*	*INSR*	*MT-CO1*	*POSTN*	*SPTA1*	
CHD2	*FGFR2*	*INTU*	*MT-RNR2*	*PPARA*	*SPTAN1*	

Table S2. A list of 144 Human CHD disease genes. The list was curated from the literature (PMIDs 29106500, 23934094, 27058611, 28991257, 27479907, 28592524, 25996639, 29089047).

ACTC1	*CITED2*	*FLT4*	*KIAA0196*	*MYH7*	*PKD2*	*SOS1*
ACVR1	*COL2A1*	*FOXC1*	*KMT2A*	*NF1*	*PLRG1*	*SRCAP*
ACVR2B	*COL9A1*	*FOXC2*	*KMT2D*	*NFATC1*	*PRDM1*	*STK4*
ADNP	*CREBBP*	*FOXH1*	*KRAS*	*NIPBL*	*PRKD1*	*STRA6*
ALDH1A2	*CRELD1*	*FOXL2*	*LBR*	*NKX2*	*PTGFRA*	*TAB2*
ALK2	*CYR61*	*FOXP1*	*LEFTY2*	*NKX2-5*	*PTPN11*	*TBX1*
ANKRD1	*DDX3X*	*GATA4*	*MAP2K1*	*NKX2-6*	*RAF1*	*TBX20*
ANKRD11	*DHCR7*	*GATA5*	*MAP2K2*	*NODAL*	*RBFOX2*	*TBX3*
B3GALTL	*DNAH11*	*GATA6*	*MCTP2*	*NOTCH1*	*RBM10*	*TBX5*
B3GAT3	*DNAH5*	*GDF1*	*MDM4*	*NOTCH2*	*RIT1*	*TDGF1*
BBS2	*DNAI1*	*GDF3*	*MED13L*	*NPHP2*	*RNF20*	*TFAP2B*
BCOR	*DVL1*	*GJA5*	*MEK1*	*NPHP3*	*ROBO1*	*TNNI3*
BMPR1A	*DYRK1A*	*GPC3*	*MESP1*	*NPHP4*	*ROR2*	*VCAN*
BRAF	*EHMT1*	*HAND2*	*MID1*	*NR1D2*	*SALL1*	*WNT5A*
CBL	*ELN*	*HAS2*	*MKKS*	*NR2F2*	*SALL4*	*ZEB2*
CCDC11	*EP300*	*HOXA1*	*MKRN2*	*NRAS*	*SEMA3D*	*ZFMP2*
CDK13	*EVC*	*HRAS*	*MKS1*	*NRP1*	*SH3PXD2B*	*ZFPM2*
CEP152	*EVC1*	*IRX4*	*MLL2*	*NSD1*	*SHOC2*	*ZIC3*
CFC1	*EVC2*	*JAG1*	*MMP21*	*PACS1*	*SHROOM3*	
CHD4	*FBN1*	*JARID2*	*MYH11*	*PDGFRA*	*SMAD6*	
CHD7	*FGFR3*	*KDM6A*	*MYH6*	*PITX2*	*SMURF1*	

Table S3. Primers used in the study

Name	5´-3´sequence
Primers for test of morpholinos	
zAdcy2a-SP-MO-test-F	GGAGTTTGAAAAGCGTCAGC
zAdcy2a-SP-MO-test-R	GGTACACCACCTGCTTCCAT
zItpr1b-SP-MO-test-F	TGTGGAGGTGGTGAGAAAGC
zItpr1b-SP-MO-test-R	AGAGGCTGTCCTGCGTTTAC
zPlcb2-SP-MO-test-F	ACGCTCTGCTTATCGACCTG
zPlcb2-SP-MO-test-R	GTCTGGTAGTTGAGGGCCAC
zAdcy2a-SP-MO2-test-F	GCCAGTCTGCAGTTCAACAT
zAdcy2a-SP-MO2-test-R	TGCCAGAATCTGCCATACCA
zPlcb2-SP-MO2-test-F	CTCTGATGAGGGAACGGCTG
zPlcb2-SP-MO2-test-R	AATCTTAATAGTGAGCGTGC
Primers for qRT-PCR	
mef2cb-F	ACTCGGACATAGTGGAGACCCTG
mef2cb-R	TTCTTTGCAGGCCGTGGTGGG
actb1-F	AGATCTTCACTCCCCTTGTTCAC
actb1-R	AAACCGGCTTTGCACATACC
rpl13a-F	TCTGGAGGACTGTAAGAGGTATGC
rpl13a-R	AGACGCACAATCTTGAGAGCAG

Table S4. Variants identified in known human CHD genes.

Gene	Variant	Mutation	Family	MAF [a]	Sub population with MAF >0.01 [b]	CADD score [c]	ClinVar interpretation (N) [ID]
ANKRD1	10-92675322-G-A	p.Ala276Val	346	0.002638	AJ (0.02086)	23.00	Benign(5);Likely benign(5);Uncertain significance(1) [45639]
CCDC11	18-47765069-C-T	p.Arg407Gln	3315	0.000025	n/a	25.70	n/a
CDK13	7-40132656-G-A	p.Val1170Met	3315	0.005165	n/a	26.10	n/a
CYR61	1-86048526-C-G	p.Ser316Cys	3540	0.004706	n/a	24.60	n/a
DNAH11	7-21600738-A-G	p.Gln311Arg	226	0.000077	n/a	14.36	n/a
	7-21760479-G-A	c.7287+5 G>A	346	0.000074	n/a	14.54	n/a
	7-21789835-G-A	c.8798-5 G>A	645	0.003759	n/a	14.13	Benign(1);Likely benign(2);Uncertain significance(1) [163114]
	7-21788220-C-G	p.Arg2845Gly	720	0.001274	n/a	16.60	Likely benign(1);Uncertain significance(1) [359658]
	7-21640338-G-T	p.Glu1015Asp	1722	0.002989	n/a	22.70	Benign (2) [238905]
DNAH5	5-13850914-C-T	p.Arg1654Gln	489	0.0002617	n/a	32.00	Uncertain significance (2) [238977]
	5-13914751-C-T	p.Val400Met	732	0.0008012	n/a	34.00	Likely benign (1) [219733]

	5-13786351-C-G	p.Glu2919Asp	1121	0.003120	n/a	15.84	Benign(2);Likely benign(1);Uncertain significance(1) [188365]
EVC2	4-5642347-G-C	p.Thr455Arg	545	0.003836	n/a	23.90	Benign(3);Likely benign(1);Uncertain significance(1) [193762]
	4-5620263-G-A	p.Ala803Val	1151	0.003752	FIN (0.01365)	16.90	n/a
FOXH1	8-145700381-C-G	p.Ser113Thr	346	0.004183	n/a	22.70	Benign/Likely benign (5) [94385]
KMT2A	11-118352769-G-A	p.Ser1325Asn	49	0.000906	n/a	19.46	n/a
KMT2D	12-49426025-G-T [d]	p.Pro4155Thr	346	0.0000281	n/a	14.07	n/a
	12-49431844-G-A [d]	p.Arg3099Cys	3505	0.00002884	n/a	24.80	n/a
MYH11	16-15797967-T-A	p.Thr1934Ser	732	0.001347	n/a	16.85	Likely benign(5);Uncertain significance(1) [201044]
MYH6	14-23866451-G-T	Leu700Ile	1710	n/a	n/a	24.20	n/a
NFATC1	18-77246538-G-A	p.Gly795Arg	645	0.003186	n/a	14.95	n/a
NOTCH2	1-120468201-A-T	p.Leu1413His	1121	0.003299	n/a	15.52	Benign/Likely benign (5)[134975]
NPHP4	1-6012895-G-A	c.675 C>T (splice)	489	0.000086	n/a	5.49	n/a
PACS1	11-66008953-G-A	Gly829Ser	1151	n/a	n/a	23.10	n/a

PDGFRA	4-55161324-C-T	p.Thr1052Met	1151	0.0001769	n/a	25.20	Uncertain significance (2) [39618]
PKD2	4-88967919-T-G	p.Phe482Cys	545	0.001984	n/a	24.90	Benign (3) [219481]
ROR2	9-94487187-C-T	p.Arg530Gln	732	0.001915	n/a	20.90	Likely benign (4) [284633]
TBX5	12-114837364-T-C	p.Ile106Val	226	0.000933	AJ (0.01119)	21.80	Likely benign (3) [213825]
VCAN	5-82868257-A-G	p.Asn3253Ser	346	0.0002088	n/a	26.10	n/a
	5-82816284-T-C	p.Val720Ala	732	0.001995	n/a	0.001	Likely benign (3) [354403]

[a] Minor allele frequency in gnomAD v2.1.1 (141,456 individuals, http://gnomad.broadinstitute.org). [b] GnomAD sub population with MAF >0.01. AJ: Ashkenazi Jewish, FIN: European (Finnish). MAF in subpopulation is shown in paranteses. [c] CADD score according to the Combined Annotation-Dependent Depletion prediction algorithm. n/a: not available. [d] None of the patients with these variants in *KMT2D* had features of Kabuki syndrome.

Table S5. Significant protein-protein interaction clusters with at least two CDGs. Statistical significance was determined by permutation analysis (k=10,000). *:P<0.05, **:P<0.01.

Cluster id.	Cluster size	Mut.Genes	p-value
cluster15	27	11	0.00331 **
cluster17	5	3	0.03872 *
cluster215	21	7	0.05427
cluster28	13	5	0.05775
cluster40	6	3	0.06705
cluster202	6	3	0.06807
cluster200	12	4	0.13646
cluster14	23	6	0.19057
cluster8	5	2	0.20428
cluster3	29	7	0.22072
cluster208	10	3	0.24048
cluster10	6	2	0.27304
cluster6	6	2	0.27311
cluster47	6	2	0.27341
cluster230	6	2	0.27429
cluster42	6	2	0.27477
cluster41	6	2	0.27558
cluster13	7	2	0.34492
cluster7	8	2	0.41017
cluster5	15	3	0.48476
cluster4	27	5	0.5071
cluster9	17	3	0.57995
cluster2	18	3	0.61678
cluster113	14	2	0.71902
cluster1	30	4	0.78055

Table S6. Rare calcium signaling gene variants shared among affected individuals in multiplex CHD families.

Family	Gene	Gene pLI [a]	Variant	Mutation [b]	MAF [c]	Sub pop. With MAF>0.01 [d]	CADD score [e]	MPC score	Heart malformations
333	*ITPR1*[e]	1.000	3-4704816-G-A	V479I	0.004560	AJ (0.01023)	22.2	1.77	PAPVR, ASDsv, VSD, COA
346	*ADCY2*[f]	1.000	5-7695981-G-A	IVS6+5 G>A	0.005025	AJ (0.01529), FIN (0.02375)	15.1	n/a	VSD
	NFAT5[g]	1.000	16-69660329-A-G	K51E	0.000042	n/a	27.0	1.95	VSD
489	*PLCB2*[h]	0.000	15-40584565-C-A	K802N	0.000004	n/a	29.7	n/a	ASD, subAS, VSD, PDA, HCM
	PLCB2[h]	0.000	15-40584570-T-A	M801L	n/a	n/a	24.9	n/a	ASD, subAS, VSD, PDA, HCM
	PLCB2[h]	0.000	15-40584571-C-A	E800D	n/a	n/a	21.1	n/a	ASD, subAS, VSD, PDA, HCM
645	*CACNA1D*[i]	1.000	3-53707750-C-T	A376V	0.00001591	n/a	32.0	2.04	ASD, VSD
702	*CACNA1S*[i]	0.000	1-201009430-G-T	P1767T	0.0002199	n/a	3.34	0.11	VSD, AVSD
732	*CACNA1H*[i]	0.756	16-1260783-G-A	IVS20-4 G>A	0.003458	AJ (0.03019)	22.9	n/a	TOF
	CACNA1H[i]	0.756	16-1270119-C-T	R2063W	0.00009303	n/a	0.07	n/a	TOF
	GRIA4[j]	0.989	11-105776016-G-C	G383R	0.000004002	n/a	26.5	n/a	TOF
1121	*CACNA1F*[i]	0.874	X-49088240-T-C	T59A	0.00005878	n/a	0.12	0.54	ASD, VSD, PVS
1151	*CACNA1S*[i]	0.000	1-201009011-C-T	S1857N	0.002224	n/a	23.3	0.17	ASD, EbA
1722	*ADCY5*[f]	0.990	3-123046510-C-G	E634D	0.004502	n/a	15.1	n/a	ASD, COA, DCM
	ITPR1[e]	1.000	3-4842276-G-A	A2352T	0.0008309	n/a	23.5	0.92	ASD, COA, DCM
2077	*CACNA1I*[i]	1.000	22-40059828-G-C	Q1193H	0.003735	AJ (0.03569)	23.7	n/a	PVS, InfPS

[a] Probability of loss of function intolerance of gene. [b] All 16 listed mutations were confirmed by Sanger sequencing. [c] Minor allele frequency in gnomAD v2.1.1 (141,456 individuals, http://gnomad.broadinstitute.org). n/a: not available in database.[c] GnomAD sub population with MAF >0.01. AJ: Ashkenazi Jewish, FIN: European (Finnish). MAF in subpopulation is shown in paranteses. [d] C score according to the Combined Annotation-Dependent Depletion (CADD) prediction algorithm [27]. [d] MPC-score: score based on regional missense constraint. [e] IP3 receptor. [f] Adenylate cyclase. [g] Transcription factor. [h] Phospholipase C. [i] Voltage Dependent Calcium Channel. [j] Glutamate receptor.

Table S7. Gene ontology term enrichment of 27 genes within the cluster shown in Figure 3A. P-values were adjusted for multiple testing.

**Gene Ontology – Molecular Function**	**p-value**
calcium-dependent protein kinase C activity (GO:0004698)	1.61E-06
calcium-dependent protein serine/threonine kinase activity (GO:0009931)	3.35E-05
calcium-dependent protein kinase activity (GO:0010857)	4.35E-05
protein kinase C activity (GO:0004697)	1.47E-04
histone kinase activity (H3-T6 specific) (GO:0035403)	9.01E-04
protein serine/threonine kinase activity (GO:0004674)	2.60E-03
ATP binding (GO:0005524)	2.99E-03
adenyl ribonucleotide binding (GO:0032559)	3.44E-03
adenyl nucleotide binding (GO:0030554)	3.57E-03
purine ribonucleoside triphosphate binding (GO:0035639)	1.03E-02
purine ribonucleoside binding (GO:0032550)	1.06E-02
purine nucleoside binding (GO:0001883)	1.07E-02
ribonucleoside binding (GO:0032549)	1.08E-02
cAMP-dependent protein kinase activity (GO:0004691)	1.10E-02
histone threonine kinase activity (GO:0035184)	1.10E-02
nucleoside binding (GO:0001882)	1.10E-02
purine ribonucleotide binding (GO:0032555)	1.18E-02
purine nucleotide binding (GO:0017076)	1.23E-02
ribonucleotide binding (GO:0032553)	1.31E-02
cyclic nucleotide-dependent protein kinase activity (GO:0004690)	1.82E-02
protein kinase activity (GO:0004672)	2.18E-02
adenylate cyclase activity (GO:0004016)	2.25E-02
carbohydrate derivative binding (GO:0097367)	3.51E-02

**Gene Ontology – Biological Process**	**p-value**
activation of phospholipase C activity (GO:0007202)	1.59E-07
positive regulation of phospholipase C activity (GO:0010863)	3.30E-07
regulation of phospholipase C activity (GO:1900274)	3.83E-07
positive regulation of phospholipase activity (GO:0010518)	1.03E-06
regulation of phospholipase activity (GO:0010517)	1.82E-06
positive regulation of lipase activity (GO:0060193)	2.14E-06
regulation of lipase activity (GO:0060191)	4.61E-06
activation of protein kinase A activity (GO:0034199)	3.76E-04
epidermal growth factor receptor signaling pathway (GO:0007173)	8.48E-04
fibroblast growth factor receptor signaling pathway (GO:0008543)	8.75E-04
regulation of body fluid levels (GO:0050878)	1.01E-03
ERBB signaling pathway (GO:0038127)	1.02E-03
cellular response to fibroblast growth factor stimulus (GO:0044344)	1.27E-03
response to fibroblast growth factor (GO:0071774)	1.38E-03
peptidyl-serine phosphorylation (GO:0018105)	2.19E-03

neurotrophin TRK receptor signaling pathway (GO:0048011)	2.37E-03
neurotrophin signaling pathway (GO:0038179)	2.46E-03
transmembrane receptor protein tyrosine kinase signaling pathway (GO:0007169)	2.51E-03
histone H3-T6 phosphorylation (GO:0035408)	2.74E-03
renal water homeostasis (GO:0003091)	2.74E-03
peptidyl-serine modification (GO:0018209)	3.18E-03
energy reserve metabolic process (GO:0006112)	3.27E-03
cellular response to glucagon stimulus (GO:0071377)	3.86E-03
activation of adenylate cyclase activity (GO:0007190)	4.52E-03
positive regulation of adenylate cyclase activity (GO:0045762)	8.41E-03
response to glucagon (GO:0033762)	8.41E-03
enzyme linked receptor protein signaling pathway (GO:0007167)	9.95E-03
synaptic signaling (GO:0099536)	1.11E-02
synaptic transmission (GO:0007268)	1.11E-02
trans-synaptic signaling (GO:0099537)	1.11E-02
multicellular organismal water homeostasis (GO:0050891)	1.48E-02
positive regulation of cyclase activity (GO:0031281)	1.72E-02
positive regulation of lyase activity (GO:0051349)	1.72E-02
water transport (GO:0006833)	1.90E-02
water homeostasis (GO:0030104)	1.99E-02
fluid transport (GO:0042044)	2.49E-02
regulation of adenylate cyclase activity (GO:0045761)	2.60E-02
histone-threonine phosphorylation (GO:0035405)	3.35E-02
positive regulation of cAMP biosynthetic process (GO:0030819)	3.87E-02
immune system process (GO:0002376)	3.91E-02
cellular response to forskolin (GO:1904322)	4.38E-02
response to forskolin (GO:1904321)	4.38E-02
regulation of cyclase activity (GO:0031279)	4.63E-02
regulation of lyase activity (GO:0051339)	4.97E-02

Table S8. Replication using WES data from 714 CHD cases and 4922 controls. The number of cases and controls with rare variants (MAF < 0.001) in any of the genes *ADCY2*, *ADCY5*, *CACNA1D*, *CACNA1H*, *CACNA1I*, *CACNA1S*, *GRIA4*, *ITPR1*, *NFAT5* and *PLCB2* was compared (see supplemental material for details). WES data was not available for *CACNA1F*.

Type of rare variant [a]	Cases	Controls	P-value [b]		Odds ratio
Protein sequence altering variants	116	877	3.18E-01	n.s.	0.89 [0.72-1.11]
Protein altering variants, MPC score >1	43	243	3.35E-01	n.s.	1.23 [0.86-1.73]
Protein altering variants, MPC score >2	21	55	3.69E-04	**	2.68 [1.53-4.54]
Protein truncating variants	3	16	7.25E-01	n.s.	1.29 [0.24-4.53]
Synonymous variants	94	630	7.65E-01	n.s.	1.03 [0.81-1.31]

[a] Variants with pathogenic effect was defined by MPC score >2. [b] Fisher's exact test. **:P<0.01. n.s.: not significant.
